# Genomic, Transcriptomic and Metabolomic Studies of Two Well-Characterized, Laboratory-Derived Vancomycin-Intermediate *Staphylococcus aureus* Strains Derived from the Same Parent Strain

**DOI:** 10.3390/antibiotics4010076

**Published:** 2015-02-04

**Authors:** Dipti S. Hattangady, Atul K. Singh, Arun Muthaiyan, Radheshyam K. Jayaswal, John E. Gustafson, Alexander V. Ulanov, Zhong Li, Brian J. Wilkinson, Richard F. Pfeltz

**Affiliations:** 1 School of Biological Sciences, Illinois State University, Normal, IL 61790, USA; E-Mails: dshatta@ilstu.edu (D.S.H.); sangatul@gmail.com (A.K.S.); amuthai@gmail.com (A.M.); drjay@ilstu.edu (R.K.J.); 2 Department of Biochemistry and Molecular Biology, Oklahoma State University, Stillwater, OK 74078, USA; E-Mail: john.gustafson@okstate.edu; 3 Roy J. Carver Biotechnology Center, University of Illinois at Urbana-Champaign, Urbana, IL 61807, USA; E-Mails: ulanov@illinois.edu (A.V.U.); lucasli@illinois.edu (Z.L.); 4 BD Diagnostic Systems, Microbiology Research and Development, Sparks, MD 21152, USA; E-Mail: richard_pfeltz@bd.com

**Keywords:** *Staphylococcus aureus*, VISA, genomics, transcriptomics, metabolomics

## Abstract

Complete genome comparisons, transcriptomic and metabolomic studies were performed on two laboratory-selected, well-characterized vancomycin-intermediate *Staphylococcus aureus* (VISA) derived from the same parent MRSA that have changes in cell wall composition and decreased autolysis. A variety of mutations were found in the VISA, with more in strain 13136p^−^m^+^V20 (vancomycin MIC = 16 µg/mL) than strain 13136p^−^m^+^V5 (MIC = 8 µg/mL). Most of the mutations have not previously been associated with the VISA phenotype; some were associated with cell wall metabolism and many with stress responses, notably relating to DNA damage. The genomes and transcriptomes of the two VISA support the importance of gene expression regulation to the VISA phenotype. Similarities in overall transcriptomic and metabolomic data indicated that the VISA physiologic state includes elements of the stringent response, such as downregulation of protein and nucleotide synthesis, the pentose phosphate pathway and nutrient transport systems. Gene expression for secreted virulence determinants was generally downregulated, but was more variable for surface-associated virulence determinants, although capsule formation was clearly inhibited. The importance of activated stress response elements could be seen across all three analyses, as in the accumulation of osmoprotectant metabolites such as proline and glutamate. Concentrations of potential cell wall precursor amino acids and glucosamine were increased in the VISA strains. Polyamines were decreased in the VISA, which may facilitate the accrual of mutations. Overall, the studies confirm the wide variability in mutations and gene expression patterns that can lead to the VISA phenotype.

## 1. Introduction

The first report of a clinical isolate of *Staphylococcus aureus* showing decreased susceptibility to vancomycin, minimum inhibitory concentration (MIC) 8 μg/mL, appeared in 1997 [1]. Since that time there have been many reports of laboratory-derived and clinically-isolated vancomycin-intermediate *S. aureus* (VISA), and these have been reviewed [2,3,4]. Despite considerable effort, the mechanism(s) underlying decreased vancomycin susceptibility is not entirely clear. Although VISA typically show a number of phenotypic traits in common, such as increased cell wall thickness, decreased autolysis and alterations in peptidoglycan structure, a wide variety of mutations and transcriptomes have been found in VISA [2,3,4]. Exposure of *S. aureus* to cell wall-active antibiotics induces the expression of a set of genes that comprises a cell wall stress stimulon [5]. Altered stress response gene expression without antimicrobial exposure, including members of the cell wall stress stimulon, is characteristic of VISA [6].

In 2000, Pfeltz *et al.* [7] reported on the characteristics of a number of VISA strains generated by *in vitro* step selection from vancomycin-susceptible *S. aureus* (VSSA) in the presence of increasing concentrations of vancomycin. Among these VSSA was heterogeneously methicillin-resistant *S. aureus* (MRSA) strain 13136p^−^m^+^ (penicillinase-negative, *mecA*-positive). This strain was originally isolated in 1960 as MRSA 13136, one of the handful of MRSA among several thousand clinical *S. aureus* isolates surveyed [8]. Strain 13136p^−^m^+^ is a penicillinase-negative derivative selected from 13136 [9]. Like the well-studied penicillinase-negative, homogeneous MRSA strain Colindale 9204 (COL), the 13136 lineage is lysogenized by prophage L54a inserted near the 3' end of the glycerol ester hydrolase gene (*geh*). The result is a truncated, inactive gene product (lipase) [10]. L54a, also known as ϕCOL, was only present in COL in a comparison of six staphylococcal genome sequences [11]. Although COL and 13136 were isolated in the same time period (1960–1964) by the Cross-infection Reference Laboratory, Colindale, London, a comparison of genome sequences between the two strains is not available. COL was isolated from a hospital in Colindale, whereas the separate survey identifying 13136 drew isolates from hospitals primarily in southeastern England, but also elsewhere within and outside of that country [8,12].

MRSA 13136p^−^m^+^ was the parent strain from which VISA 13136p^−^m^+^V5 was derived, and subsequently VISA 13136p^−^m^+^V20 was then derived from 13136p^−^m^+^V5. The MICs of the VISA strains were 8 and 16 μg/mL for 13136p^−^m^+^V5 and 13136p^−^m^+^V20, respectively [7]. Strain 13136p^−^m^+^V5 was isolated after two selection cycles in one week and strain 13136p^−^m^+^V20 after a total of five cycles of selection across five weeks. Each selection cycle consisted of growth in liquid medium that was inoculated onto solid medium containing the same concentration of vancomycin. The 64 µg/mL minimum bactericidal concentration (MBC) of strain 13136p^−^m^+^V20 was significantly higher than that of 13136p^−^m^+^V5 (16 µg/mL) or the parent strain (8 µg/mL). These strains have been extensively studied for their cell wall-related properties [7,13]. The VISA strains grew slower than the VSSA parent [7]. Muropeptide analysis showed an increase in muropeptides with a single L-alanine residue in the interpeptide bridge in the two VISA strains compared to the VSSA parent strain. Cell wall teichoic acid content was significantly decreased in the VISA strains, in strain 13136p^−^m^+^V20 in particular, as judged by cell wall phosphorus content. The VISA strains showed decreased whole cell autolysis, decreased lysostaphin susceptibility and decreased isolated crude cell wall autolytic activity. Differences were noted in the amount and profile of peptidoglycan hydrolase activities in the VISA strains [13]. *atl*, the gene encoding two major *S. aureus* autolysins [14], showed significantly reduced expression in 13136p^−^m^+^V20 compared to 13136p^−^m^+^.

We have used these strains to further probe the mechanism(s) of decreased vancomycin susceptibility by a combination of genomic, transcriptomic and metabolomic approaches. Metabolomics typically involves the comparative analysis of small, cellular organic compounds of a molecular weight less than 1000 daltons [15]. A metabolome is a direct reflection of the physiological status of a cell and thus is another approach to understanding cell function. VISA strains are believed to be activated for cell wall synthesis [16] and to display impaired acetate catabolism [17]. A recent metabolomic analysis of two series of VISA isolates indicated alterations to a wide range of central metabolic pathway intermediates, but did not elucidate the underlying mechanism of decreased vancomycin susceptibility [18].

## 2. Results

### 2.1. Mutations in Strains 13136p^−^m^+^V5 and 13136p^−^m^+^V20 Compared to 13136p^−^m^+^

The mutations present in the two VISA strains are summarized in Table 1. A total of nine single-nucleotide polymorphisms (SNPs) across eight genes that resulted in protein amino acid changes were detected in strain 13136p^−^m^+^V5 and 16 SNPs across nine genes in 13136p^−^m^+^V20, relative to VSSA parent strain 13136p^−^m^+^. Although the MIC of 13136p^−^m^+^V20 was only double that of 13136p^−^m^+^V5, strain 13136p^−^m^+^V20 was subjected to three more selection cycles than 13136p^−^m^+^V5, and this was reflected in the higher number of SNPs. Six of the nine 13136p^−^m^+^V5 SNPs were also present in 13136p^−^m^+^V20, but three were not.

**Table 1:** Mutations resulting in protein amino acid changes present in VISA strains 13136p^−^m^+^V5 and 13136p^−^m^+^V20 (yellow cells) following in-vitro passage-selection from VSSA parent 13136p^−^m^+^.

	SNP	Amino acid change	Locus	Gene Name	Known or Predicted Gene Product	Mutation Present In:		Function of Predicted Gene Product Based on Published Studies and Similarities to Other Proteins, and Known Stress-Response Associations [references]
						13136p^−^m^+^V5	13136p^−^m^+^V20	
223	G→G	K→E	SACOL1005	*pepF*	Oligoendopeptidase F	+	-	Cytoplasmic endopeptidase releasing amino acids from internalized peptides; involved in protein turnover [19]
143	C→T	A→V	SACOL1231	*stp1*	Eukaryotic-like serine / threonine phosphatase	+	-	Influences the regulation of virulence, cell wall structure, autolysis, and susceptibility to some cell wall-active antibiotics [20]
554	G→A	S→F	SACOL1600	*comGB*	ComGB, competence protein	+	-	A membrane protein associated with the SOS response that transports exogenous DNA into the Gram-positive cell [21]
126	A→C	E→D	SACOL0593	*fusA*	Translation elongation factor G	-	+	Protein synthesis roles in tRNA translocation during elongation and post-termination ribosome dissociation; upregulated by acid adaptation [22,23]
121	G→T	G→C	SACOL0593			+	+	
6596	G→A	A→V	SACOL2150	*fmtB*	FmtB protein	+	+	β-lactam resistance-related surface protein with cell wall anchoring and spanning domains, putatively involved in cell wall biosynthesis, cell adhesion, biofilms [6]
171	C→A	M→I	SACOL2217	*infA*	Translation initiation factor IF-1	+	+	RNA-binding protein that binds the 30S ribosomal subunit, required for correct translation initiation, and downregulated by cell wall-active antibiotics [5,24]
311	A→T	Y→F	SACOL1319	*glpF*	Glycerol uptake facilitator protein	+	+	Housekeeping gene product that transports glycerol or small uncharged molecules into the cell; down-regulated by oxidative stress [25]
353	G→C	G→P	SACOL0339	*ssb1*	Prophage L54a single-stranded DNA-binding protein	+	+	One of a family of proteins that bind with high affinity to ssDNA intermediates during DNA replication, recombination and repair [26,27,28]
352	G→C	G→P	SACOL0339			+	+	
350	A→C	N→P	SACOL0339			-	+	
349	A→C	N→P	SACOL0339			-	+	
345	A→C	Q→P	SACOL0339			-	+	
344	A→C	Q→P	SACOL0339			-	+	
329	A→C	K→T	SACOL0339			-	+	
670	C→T	L→F	SACOL0810	*tarO*	glycosyl transferase, group 4 family protein (TarO)	-	+	Teichoic acid synthesis enzyme; inactivation increases autolysis rates, reduces β-lactam resistance, and disrupts sepatation and cell separation [29]
11	G→A	A→V	SACOL1495	*dinG*	Damage inducible gene G (DinG)	-	+	3'→5' ssDNA and ssRNA exonuclease; proposed functions: R-loop resolution, other unspecified recombination repair systems, anitviral defense [30]
191	C→T	C→Y	SACOL1690	*apt*	Adenine phosphoribosyl transferase	-	+	Enzyme in an adenine recycling pathway upregulated by acid adaptation, the stringent response, and in some organisms polyamine metabolism [23,31]
574	A→C	F→V	SACOL2451	none	Amino acid ABC transporter binding protein	-	+	Homolog of OpuBC, the glycine betaine/choline binding lipoprotein of an osmoprotectant uptake system [32]

A total of 12 genes were associated with 19 protein amino acid changes in one or both VISA (Table 1). Five of these genes encode products that interact with nucleic acids in protein synthesis or are associated with DNA damage/SOS responses: *comGB*, *fusA*, *infA*, *ssb1* and *dinG* [21,22,23,24,26,27,28,30]. Three more are housekeeping genes encoding products involved in central intermediary metabolism: *pepF* encodes an endopeptidase that hydrolyzes internalized peptides to provide nutrients or endogenous peptides for protein turnover [19]; *glpF* encodes a glycerol transporter (carbon and energy acquisition) [25]; and *apt* encodes an enzyme in a purine salvage pathway catalyzing the production of AMP from adenine and phosphoribosyl pyrophosphate [23,31]. *apt* is associated with the stringent response in other Gram-positive bacteria [29,33,34,35]. The *fmtB* and *tarO* gene products are cell envelope proteins involved in cell wall biosynthesis, β-lactam resistance and virulence [6,29]. *fmtB* is located downstream of *glmM* and is also known as *mrp* and *sasB* [36]. FmtB has a C-terminal LPXTG cell wall anchoring motif [37]. The indirect effect of FmtB on methicillin resistance can be relieved by increasing the production of the cell wall precursor glucosamine-l-phosphate [38]. *tarO*, also known as *tagO*, *llm* and *farO*, catalyzes the initial step of cell wall teichoic acid synthesis. Locus SACOL2451 encodes a putative membrane protein, a homolog of OpuBC, the permease component of an ABC-type osmoprotectant transport system [32].

The final mutated gene, *stp1*, encodes a eukaryotic-like serine-threonine phosphatase and is located in tandem with its cognate serine-threonine kinase gene, *stk1* [39]. These two enzymes comprise a two-component regulatory system that plays important roles in bacterial signal transduction [20], in addition to roles in cell wall structure and autolysis [39,40]. Mutations in *stp1* have previously been associated with laboratory-selected glycopeptide resistance, along with cell wall alterations characteristic of the VISA phenotype [40,41,42,43]. Passalacqua *et al.* [42] reported reduced autolytic activity in addition to decreased susceptibility to vancomycin, daptomycin and linezolid due to an *stp1* mutation. Additionally, Cameron *et al.* [43] demonstrated that the deletion of this gene alone is sufficient to slightly reduce vancomycin susceptibility. A eukaryotic-like serine-threonine phosphatase-kinase pair regulates an osmotic stress sensory system in *Mycobacterium tuberculosis* that leads to an adaptive response involving modifications to cell wall structure and virulence factor production [44]. Eleven of the 12 genes listed in Table 1, all except *pepF*, can be linked to stress response either by expression change or gene product function.

### 2.2. Comparison of Transcriptional Profiles between 13136p^−^m^+^, 13136p^−^m^+^V5 and 13136p^−^m^+^V20

**Figure 1:**
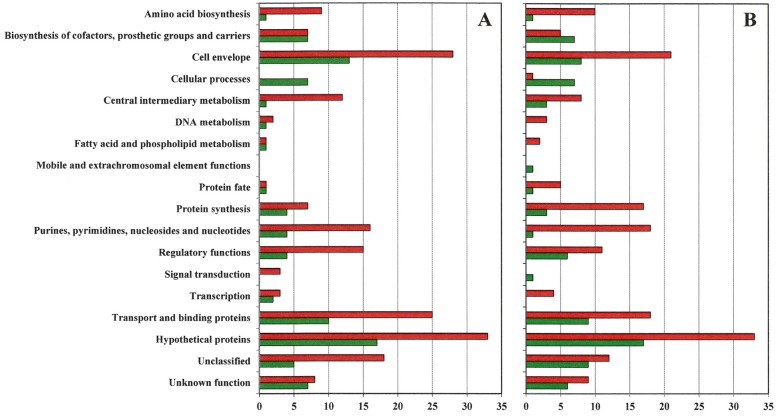
Gene expression patterns. The number of genes by functional group upregulated (green bars) and downregulated (red bars) at least two-fold in VISA 13136p^−^m^+^V5 (**A**) and 13136p^−^m^−^V20 (**B**) relative to gene expression in vancomycin-susceptible *S. aureus* (VSSA) parent 13136p^−^m^+^.

Gene expression differences in VISA 13136p^−^m^+^V5 and 13136p^−^m^+^V20 relative to VSSA progenitor strain 13136p^−^m^+^ are listed in Supplementary Tables S1 and S2. These changes are presented graphically by gene functional groupings in Figure 1. Supplementary Table S3 contains the data used to generate Figure 1. For both VISA, approximately twice as many gene expression differences *versus* VSSA 13136p^−^m^+^ were downregulated as were upregulated. Unfortunately, but not unexpectedly, the gene functional group with the largest number of changes was hypothetical functions, and along with the unclassified and unknown function categories accounted for 91 of the 338 genes (27%) with expression changes in VISA strains. Among genes with assigned functions, seven functional groups each had at least 5% of the genes each with expression changes in VISA *vs.* VSSA 13136p^−^m^+^: cell envelope (48 genes, or 14%), transport and binding proteins (39 genes, or 12%), purines, pyrimidines, nucleosides and nucleotides (28 genes, or 8%), protein synthesis (27 genes, or 8%), regulatory functions (24 genes, or 7%), and biosynthesis of cofactors, prosthetic groups and carriers (18 genes) and central intermediary metabolism (16 genes) with 5% each. Together these eight functional groups accounted for 86% of the genes represented in Figure 1. Downregulation was the clear trend for all of these functional groups, except biosynthesis of cofactors, prosthetic groups and carriers, where the numbers of down- and up-regulation were comparable.

Supplementary Table S3 presents the gene expression change concordance between VISA13136p^−^m^+^V5 and 13136p^−^m^+^V20. Approximately the same number of genes were upregulated in each VISA strain, 83 in VISA13136p^−^m^+^V5 and 80 13136p^−^m^+^V20. Of these, 53%–55% of those upregulated in one VISA were also upregulated in the other VISA strain. Approximately the same number of genes were also downregulated in each VISA strain, 187 in VISA13136p^−^m^+^V5 and 177 in 13136p^−^m^+^V20. Expression concordance was greater for underexpression patterns, as 70%–73% of genes downregulated in one VISA were also downregulated in the other VISA strain. As shown in Figure 1, VISA 13136p^−^m^+^V5 had noticeably more expression changes than 13136p^−^m^+^V20 among the cell envelope (41 *vs.* 29) and transport and binding protein (11 *vs.* 20) genes and fewer than 13136p^−^m^+^V20 among protein fate (two *vs.* six) and protein synthesis (11 *vs.* 20) genes, with downregulated genes outnumbering upregulated genes at least 2:1 for each VISA and these functional groups, except the 1:1 ratio for the two 13136p^−^m^+^V5 protein fate expression changes. Total numbers of regulatory function gene expression changes were comparable between the two VISA, at 19 for VISA13136p^−^m^+^V5 and 17 for 13136p^−^m^+^V20, but the ratio of the number of upregulated to downregulated genes varied between the strains at 4:15 and 6:11, respectively.

Although overall gene expression trends were similar between the two VISA *versus* their progenitor VSSA, specific patterns within these data were not consistent between the two comparisons. For example, 13 of the 16 ribosomal proteins listed in Supplementary Table S1 were downregulated for 13136p^−^m^+^V20, with three unchanged, compared to 13136p^−^m^+^. In contrast, four were downregulated and 12 unchanged for the 13136p^−^m^+^V5 to 13136p^−^m^+^ comparison. However, for the expression of 18 *pur* and *pyr* genes associated with central intermediary biosynthetic pathways, 12 were downregulated and six unchanged in 13136p^−^m^+^V5, and 15 were downregulated and three unchanged in 13136p^−^m^+^V20. For either ribosomal protein or *pur* and *pyr* genes, when expression was unchanged in one VISA, it was always downregulated in the other VISA *versus* 13136p^−^m^+^. Genes encoding secreted proteases were generally downregulated in both VISA (*sec3*, *sspA*, *sspB2* and *sspC*), as were *cap* genes encoding capsular polysaccharide biosynthesis, but the pattern of expression changes was inconsistent between the two VISA. The expression of genes encoding cell surface protein, regulatory, biofilm-related and cell wall metabolism gene products was more variable, with more frequent upregulation and greater ranges of expression fold-change values. A large number of genes previously identified as associated with the VISA strain, including members of the cell wall stress stimulon, showed expression changes in one or both VISA *versus* 13136p^−^m^+^. These include *vraR* and *prsA*, which are overexpressed in both VISA and are part of the cell wall stress stimulon [5], as well as penicillin binding proteins. McAleese *et al.* [6] previously reported overexpression of the cell wall stress stimulon in VISA.

Three genes, which were altered in expression, as determined by microarray analysis, were validated using qRT-PCR. The expression ratios of *cmk*, *agrA* and *ddh* were shown to be in good agreement with microarray results (data not shown).

### 2.3. Genes with at Least One Eight-Fold Expression Change among VISA and VSSA

Table 2 is the subset of genes from Supplementary Table S1 with large expression differences among the three strains, as defined by at least one expression change eight-fold or greater in magnitude (≥8-fold) between either VISA and the progenitor VSSA. Supplementary Figure S1 is a graphical representation of the data in Table 2. In Table 2 genes are sorted by attributes related to the VISA phenotype instead of generic functional groupings. Additionally, the Table 2 V20-V5 column indicates fold-change expression differences between VISA 13136p^−^m^+^V20 and 13136p^−^m^+^V5. A total of 60 changes ≥8-fold were present between the three strains across 52 different genes. The hypothetical/ unclassified/unknown function gene functional group accounted for 21 (40%) of the 52 genes with ≥8-fold changes, followed by 11 cell envelope genes (21%), four genes each (8%) in regulatory functions and biosynthesis of cofactors, prosthetic groups and carriers and three genes each (6%) for purines, pyrimidines, nucleosides, nucleotides and transport and binding proteins; together, these groups accounted for 89% of the ≥8-fold change gene subset. This pattern differed from that in Figure 1 and Supplementary Table S2 in that some functional groups had no genes among the ≥8-fold change gene subset, including the amino acid biosynthesis, transcription and, most notably, the protein synthesis groups. The general central intermediary metabolism functional group had two genes in the ≥8-fold change gene subset, just below 4%, but comparable to the total in Supplementary Table S1.

**Table 2:** Expression patterns and assigned functional roles for genes with at least one ≥ 8-fold expression increase or decrease (yellow cells) between VSSA 13136p^-^m^+^ and VISA or VISA 13136p^−^m^+^V5 and 13136p^−^m^+^V20.

Locus ID	Gene	Protein	13136p^−^m^+^V5 *vs*. VSSA	13136p^−^m^+^V20 *vs*. VSSA	VISA V20 *vs*. VISA V5	VISA-Related Categories	Stress Response Association	Gene and Protein Functional Role Comments [references]
SAV2009	*sec3*	enterotoxin typeC3	−24.4	−21.5	2.9	Virulence Factor-Associated		Secreted exotoxin, same as gene *sea* in MRSA strain COL [11]
SACOL0907	*seb*	staphylococcal enterotoxin B	−17.2	−18.3	−1.1		Nutritional	Secreted cytotoxin upregulated during the stringent response [45]
SACOL1871	*epiG*	epidermin immunity protein F	−11.2		11.2		Nutritional	Virulence factor upregulated during the stringent response [45]
SAV2472	NA	short chain dehydrogenase	−8.5	−5.2	3.3			Capsular biosynthesis enzyme from the SDR protein super-family [46]
SAV0372	NA	predicted PepSY family membrane peptidase propeptide	−4.1	−10.5	−6.4			Unknown function but likely to have a protease inhibitory function based on homology to peptidase propeptides
SAV1046	*sspC*	cysteine protease	−3.4	−8.6	−5.2			Secreted virulence factor Staphostatin B that inhibits *sspB2*-encoded serine protease Staphopain B activity [47]
SACOL2026	*agrA*	accessory gene regulator protein A	−2.8	−9	−6.2			Response regulator of the agr operon which generally upregulates secreted proteins and downregulates cell surface proteins [48]
SACOL0096	*sarS*	staphylococcal accessory regulator S		8.9	8.9		Nutritional	SarA global regulator family that generally upregulates virulence factor genes; sarS is downregulated by *agr* and upregulated by Rot and during the stringent response [45,48,49]
SAV0111	*spa*	Immunoglobulin G binding protein A precursor		11.5	11.5		General	Cell surface adhesion protein upregulated by heat shock and downregulated by cell wall-active antibiotics [5,47,50]
SAV1764	*rot*	repressor of toxins Rot		12.6	12.6	Virulence Factor-Associated		Homolog of the SarA transcriptional regulator that often exerts effects opposite of *agr* [48]
SAV0320	*geh*	glycerol ester hydrolase	2.7	12	9.3			*rot*-regulated lipase translated as an inactive, truncated form due bacteriophage L54a integration [10,49]
SAV2637	*aur*	zinc metalloproteinase aureolysin	8.2		−8.2			Secreted virulence factor [51]
SAV2667	*icaD*	intercellular adhesion protein D	15.9		−15.9		Oxidative	Cell surface virulence factor downregulated by oxidative stress [25]
SACOL2689	*icaA*	intercellular adhesion protein A	25.2		−25.2		Oxidative	Cell surface virulence factor downregulated by oxidative stress [25]
SAS0236	*scdA*	cell wall metabolism protein ScdA	−2	9.5	11.5	Cell Wall Metabolism	Oxidative	ScdA is involved in peptidoglycan cross-linking and cell division, and upregulated by oxidative stress [25]
SACOL0034	*mecR1*	methicillin-resistance MecR1 regulatory protein	38.5		−38.5		Cell Wall-Active Antibiotics	Integral membrane metalloprotease acting as a beta-lactam sensing signal transducer [52]
SAV0041	*mecA*	penicillin binding protein 2A	48.5		−48.5		Nutritional & Cell Wall-Active Antibiotics	Alternative PBP imparting *mec*-mediated resistance to beta-lactam antibiotics
SACOL2147	NA	transcriptional antiterminator, BglG family/DNA-binding protein	−12	−4.4	7.6	Central Intermediary Metabolism	Oxidative	Regulatory functions; downregulated by oxidative stress [25]
SACOL1573	NA	integrase/recombinase, core domain family	−10.6	−12.1	−1.5		DNA Damage	Pseudogene not located within a prophage or pathogenicity island
SAV2328	NA	dehydrogenase	−9.9	−4.4	5.5			Unknown function; from the SDR protein super-family [53]
SAV2182	*asp23*	alkaline shock protein 23	−9.8	−19.7	−9.9		Nutritional & General	σ^B^-regulated general stress response gene upregulated in VISA, by alkaline or heat shock, and during the stringent response [45,50,54]
SACOL1114	NA	Mn2+/Fe2+ transporter, NRAMP family	−9.5	−9.4	0.1		Oxidative & Nutritional	Gene upregulated by oxidative stress - Mn2+ and Fe2+ are important for oxidative stress resistance, and Mn may have a role in virulence related competition with hosts for limited nutrient [25,55]
SAV1074	*purD*	phosphoribosylamine-glycine ligase	−9.5	−2.3	7.2		Nutritional	Purine ribonucleotide biosynthesis gene downregulated during the stringent response [45]
SAV1072	*purN*	phosphoribosylglycinamide formyltransferase	−9.5	−2.5	7			Purine ribonucleotide biosynthesis gene [56]
SAV2185	NA	glycine betaine transporter opuD homolog	−9.2	−14.5	−5.3		Osmotic & General	The *opuCABCD* operon is upregulated by osmotic stress and part of the general stress response [57]
SAS0678	NA	glutamine amidotransferase class-I protein	−8.6	−5.4	3.2			Subunit of anthranilate synthase, an enzyme from the glutamate-consuming folate biosynthetic pathway
SACOL0872	NA	OsmC/Ohr family protein	−8.5	−5.7	2.8		Osmotic	Membrane protein of unknown function induced by osmotic stress
SAV1071	*purM*	phosphoribosylaminoimidazole synthetase	−8.4	−2.2	6.2		Nutritional	Purine ribonucleotide biosynthesis gene downregulated during the stringent response [45]
SACOL0630	NA	amino acid permease	−6	−9.5	−3.5			Transmembrane amino acid transporter protein
SACOL2428	*bioD*	dethiobiotin synthase		8.7	8.7			Biotin biosynthesis enzyme; competition for biotin may play an important role in phagosome escape [58]
SACOL0032	*maoC*	(R)-specific enoyl-CoA hydratase	23.7		−23.7			Amino acid degradation enzyme in aerobic phenylalanine/phenylacetate catabolism [59]
SACOL0866	NA	hypothetical protein	−12.9	−3.8	9.1	Hypothetical Proteins		
SAR0592	NA	hypothetical protein	−12.3	−15.8	−3.5			
SAV0823	NA	hypothetical protein	−11.3	−3.1	8.2			
SAR2275	NA	hypothetical protein	−9.8	−20.4	−10.6			
SACOL2547	NA	hypothetical protein	−9.5	4.2	13.7			
SACOL2720	NA	hypothetical protein	−9.4	−7	2.4			
SAS2396a	NA	hypothetical protein	−8.3	−6.4	1.9			
SAV2565	NA	hypothetical protein	−8.2	−2.3	5.9			
SAS2047	NA	hypothetical protein	−8.1	−3.9	4.2			
SACOL2174	NA	hypothetical protein	−7.4	−20.2	−12.8			
SACOL1679	NA	hypothetical protein	−7.3	−10.3	−3			
SACOL2175	NA	hypothetical protein	−6.8	−17.3	−10.5			
SACOL1680	NA	hypothetical protein	−6.3	−8.4	−2.1			
SAV2474	NA	hypothetical protein	−5.5	−10.8	−5.3			
SACOL0912	NA	hypothetical protein	−5.4	−8.3	−2.9			
SACOL1574	NA	hypothetical protein	−5.4	−8.6	−3.2			
SACOL0908	NA	hypothetical protein	−3.8	−8.8	−5			
SAS0281	NA	hypothetical protein	10	3.6	−6.4			
SACOL0625	NA	hypothetical protein	11.2	2.5	−8.7			
SACOL0067	NA	hypothetical protein	12.6	5	−7.6			
SAV2556	NA	hypothetical protein	12.8	3.6	−9.2			

Five genes in the ≥8-fold change subset that encode regulatory proteins (*agrA*, *sarS*, *rot*, *mecR1* and locus SACOL2147) all had very different expression patterns between the two VISA *versus* parent VSSA3136p^−^m^+^. The only two genes with ≥8-fold expression changes between both VISA and the parent VSSA and between the two VISA themselves were locus SAR2275, which encodes a hypothetical protein, and *asp23*, whose gene product is the σ^B^-regulated alkaline shock protein 23. This gene is downregulated by cell wall-active antibiotics and often upregulated during the general stress response and many specific stress responses, but its function is not known [5].

Genes with ≥8-fold expression changes were grouped into four VISA-related categories in Table 2: virulence factor-associated (14 genes, 12 up- and 12 down-regulated changes), cell wall metabolism (three genes, four up- and two down-regulated changes), central intermediary metabolism (14 genes, three up- and 18 down-regulated changes) and hypothetical proteins (21 genes, seven up- and 25 down-regulated changes). Of the 31 genes that did not encode hypothetical functions, 17 have one or more known stress response associations that are listed in the Table 2 Stress Response Association column. Eight genes were associated with nutritional stress (stringent response), five with oxidative stress, three with the general stress response, two each with osmotic and cell wall-active antibiotic stresses, and one with DNA damage response (recombination repair function and/or SOS response).

Ten of the ≥8-fold expression changes were upregulations (29%), and 24 were downregulations (71%) for the 13136p^−^m^+^V5 *versus* VSSA 13136p^−^m^+^ comparison. These figures were six and 20 (23% and 77%) and 10 and 13 (43% and 57%) for the 13136p^−^m^+^V20 *versus* VSSA 13136p^−^m^+^ and 13136p^−^m^+^V20 *versus* 13136p^−^m^+^V5 comparisons, respectively.

### 2.4. Metabolomics

Ninety-eight total metabolites were identified in VSSA 13136p^−^m^+^ and its two VISA derivatives, 13136p^−^m^+^V5 and 13136p^−^m^+^V20. Twenty-four were in the amines and polyamines metabolite class, 25 were amino acids, 26 were polar organic acids and the remaining 23 were sugars. The concentrations of 43 metabolites were increased and 42 were decreased in 13136p^−^m^+^V5 cells compared to the parent strain 13136p^−^m^+^ (12 were unchanged). In 13136p^−^m^+^V20 cells, the concentrations of 47 metabolites were increased and 48 were decreased compared to their concentrations in the parent strain 13136p^−^m^+^ (only two were unchanged). Thirteen metabolites were increased in both VISA and twelve others were decreased in both VISA compared to the parent VSSA. The relative concentrations of the identified metabolites in the three strains are shown in Table 3, which also contains calculated fold-changes for both VISA *versus* parent VSSA relative concentrations, as well as fold-changes for 13136p^−^m^+^V20 compared to its immediate passage-selection predecessor, 13136p^−^m^+^V5. In 13136p^−^m^+^V20 cells, the concentrations of 46 metabolites were increased, and 48 were decreased compared to their concentrations in predecessor 13136p^−^m^+^V5 13136p^−^m^+^ (four relative concentrations were unchanged).

Supplementary Figure S2 is a graphical presentation by metabolite class of the fold-change of all 98 detected metabolites’ relative concentrations for 13136p^−^m^+^V5 in comparison to the parent VSSA, 13136p^−^m^+^V20 in comparison to the parent VSSA, and 13136p^−^m^+^V20 in comparison to 13136p^−^m^+^V5. In general, a decrease in sugars was evident in strain 13136p^−^m^+^V20 *versus* its VISA predecessor 13136p^−^m^+^V5. Relative concentrations of 10 sugars increased and 12 decreased (one unchanged) in 13136p^−^m^+^V5 *versus* the parent VSSA, but the tallies were only five increased and 18 decreased for the 13136p^−^m^+^V20 *versus* parent VSSA and five increased and 16 decreased in 13136p^−^m^+^V20 in comparison to 13136p^−^m^+^V5. Most of the increased relative concentrations of sugars in 13136p^−^m^+^V20 in comparison to 13136p^−^m^+^V5 were still below those in VSSA 13136p^−^m^+^. In contrast, the relative concentrations of 15 amino acids increased and eight decreased in 13136p^−^m^+^V5 in comparison to 13136p^−^m^+^, 11 increased and 13 decreased in 13136p^−^m^+^V20 in comparison to 13136p^−^m^+^, and 12 increased and 11 decreased in 13136p^−^m^+^V20 in comparison to 13136p^−^m^+^V5.

**Table 3:** The 98 metabolites identified by metabolomic analyses, relative concentrations, and fold-change differences between strains. Empty cells: no changes (one-fold changes); ND: Not Detected, with values of 0.01 used for < and > fold-change estimates. Metabolites within each class sorted alphabetically.

Metabolite Class	Metabolite	Metabolite relative concentration per 10 mg dry weight (mean ± SD)			Metabolite Relative Concentration Fold-Change		
		VSSA 13136p^−^m^+^	13136p^−^m^+^V5	13136p^−^m^+^V20	VSSA → V5	VSSA → V20	V5 → V20
**Amines & Polyamines**	2-Amino-4,6-dihydroxypyrimidine	1.4 ± 0.2	1.9 ± 0.4	1.3 ± 0.3	1.4	−1.1	−1.4
	4,5-Dimethyl-2,6-hydroxypyrimidine	0.3 ± 0.1	0.3 ± 0.0	0.2 ± 0.0		−1.5	−1.5
	5-Methylthioadenosine	2.7 ± 0.2	2.4 ± 0.6	4.6 ± 1.0	−1.3	1.7	1.9
	Adenine	181.5 ± 21.5	138.5 ± 27.2	74.4 ± 5.5	−1.3	−2.4	−1.9
	Adenosine	125.0 ± 21.5	300.9 ± 94.4	353.6 ± 10.3	2.4	2.8	1.2
	Adenosine-5-monophosphate	ND	ND	5.7 ± 1.2		>100	>100
	Cytosine	5.5 ± 0.6	5.7 ± 1.7	3.3 ± 0.3		−1.7	−1.7
	Dihydroorotic acid	ND	3.8 ± 0.7	171.8 ± 27.9	>100	>100	45.2
	Ethanolamine	2.7 ± 0.7	4.0 ± 0.8	1.4 ± 0.1	1.5	−2	−2.9
	Glucosamine	ND	6.9 ± 1.5	129.4 ± 30.5	>100	>100	18.8
	Guanine	16.2 ± 1.2	2.7 ± 0.8	1.7 ± 0.3	−5	−10	−1.6
	Guanosine	10.9 ± 2.3	ND	42.7 ± 8.6	<−100	3.9	>100
	Hydroxylamine	1.0 ± 0.1	4.3 ± 0.8	1.9 ± 0.2	4.3	1.9	−2.3
	Hypoxanthine	ND	ND	0.1 ± 0.0		>10	>10
	Inosine	ND	ND	3.3 ± 0.7		>100	>100
	Nicotinamide	19.0 ± 1.7	13.0 ± 1.6	25.9 ± 1.1	−1.4	1.4	2
	Nicotinic acid	4.0 ± 0.9	0.5 ± 0.1	9.5 ± 1.5	−8	2.4	19
	Orotic acid	1.7 ± 0.1	1.5 ± 0.1	38.7 ± 1.3	−1.1	23	25.8
	Putrescine	63.3 ± 3.7	63.5 ± 6.5	11.8 ± 1.8		−5.4	−5.4
	Spermidine	42.1 ± 5.7	4.5 ± 0.5	3.7 ± 0.6	−9.4	−11.4	−1.2
	Thymine	15.0 ± 2.4	4.4 ± 1.1	3.4 ± 0.7	−3.4	−4.4	−1.3
	Uracil	23.7 ± 2.1	4.4 ± 0.7	6.5 ± 0.8	−5.4	−3.6	1.5
	Urea	5.6 ± 0.5	11.4 ± 2.2	3.7 ± 0.6	2	−1.5	−3.1
	Uridine	9.3 ± 1.0	57.6 ± 7.4	13.2 ± 0.9	6.2	1.4	−4.4
**Amino Acids**	4-hydroxyproline	5.5 ± 0.6	6.3 ± 1.7	3.9 ± 1.1	1.1	−1.4	−1.6
	Alanine	498.0 ± 47.1	525.3 ± 67.1	493.2 ± 41.0	1.1		−1.1
	Asparagine	18.9 ± 3.0	36.8 ± 4.5	14.7 ± 2.5	1.1	−1.3	−2.5
	Aspartic acid	975.4 ± 127.0	2203.8 ± 446.5	2303.0 ± 397.1	2.3	2.4	
	Cystathionine	4.5 ± 0.8	ND	ND	<−100	<−100	
	Glutamic acid	20.5 ± 4.0	284.7 ± 76.8	308.5 ± 36.4	14	15	1.1
	Glycine	33.8 ± 10.3	45.7 ± 6.2	109.5 ± 9.8	1.4	3.2	2.4
	Homocysteine	1.5 ± 0.3	3.7 ± 0.8	5.7 ± 1.0	2.5	3.8	1.5
	Homoserine	1.0 ± 0.2	ND	0.6 ± 0.1	<−100	−1.7	>100
	Isoleucine	26.5 ± 3.5	36.0 ± 7.1	88.9 ± 9.9	1.4	3.4	2.4
	Leucine	118.1 ± 24.2	330.4 ± 40.9	451.9 ± 68.2	2.8	3.8	1.4
	Lysine	354.5 ± 39.9	276.8 ± 20.6	121.9 ± 27.5	−1.3	−2.9	−2.3
	Methionine	10.4 ± 2.1	11.7 ± 2.8	8.5 ± 1.2	1.1	−1.2	−1.4
	N-Acetylglutamic acid	ND	ND	25.6 ± 1.5		>100	>100
	O-Acetyl-serine	5.4 ± 1.3	1.0 ± 0.2	ND	−5.4	<−100	<−100
	Ornithine	30.8 ± 4.8	16.6 ± 3.1	1.2 ± 0.2	−2	−26	−13.8
	Phenylalanine	165.9 ± 29.1	237.0 ± 30.3	116.5 ± 9.3	1.4	−1.4	−2
	Proline	10.9 ± 1.5	154.6 ± 30.0	239.8 ± 8.0	14	22	1.6
	Pyroglutamic acid	1017.3 ± 159.3	1017.6 ± 136.4	1659.0 ± 164.2		1.6	1.6
	Serine	11.5 ± 1.3	7.2 ± 1.4	9.5 ± 2.9	−1.6	−1.2	1.3
	Threonine	52.8 ± 10.6	20.0 ± 8.5	7.8 ± 1.2	−2.6	−6.8	−2.6
	Tryptophan	29.7 ± 4.8	8.5 ± 1.3	11.9 ± 3.0	−3.5	−2.5	1.4
	Tyrosine	33.6 ± 7.4	40.8 ± 5.7	26.3 ± 2.2	1.2	−1.3	−1.6
	Valine	63.1 ± 14.3	152.9 ± 25.8	170.6 ± 35.3	2.4	2.7	1.1
	β-Alanine	57.4 ± 7.6	272.3 ± 42.6	95.1 ± 12.9	4.7	1.7	−2.9
**Polar Organic Acids**	2-Hydroxyglutaric acid	6.1 ± 0.4	2.5 ± 0.3	2.8 ± 0.2	−2.4	−2.2	1.1
	2-Phosphoglycerate	14.6 ± 2.0	7.5 ± 1.9	3.0 ± 0.6	−2	−5	−2.5
	3-Hydroxybenzoic acid	0.3 ± 0.0	0.4 ± 0.1	0.5 ± 0.0	1.3	1.7	1.3
	3-Phosphoglycerate	242.6 ± 45.1	62.7 ± 10.9	40.1 ± 7.5	−3.9	−6	−1.6
	Aminomalonic acid	0.6 ± 0.1	0.21 ± 0.1	0.7 ± 0.1	2.9	1.2	3.3
	Benzoic acid	1.6 ± 0.1	1.9 ± 0.2	1.6 ± 0.4	1.2		−1.2
	cis-Aconitic acid	ND	2.1 ± 0.2	ND	>100		<−100
	Citramalic acid	14.8 ± 2.7	19.1 ± 4.1	27.3 ± 5.4	1.3	1.8	1.4
	Citric acid	15.6 ± 2.0	243.7 ± 22.6	35.1 ± 8.4	16	2.3	−6.9
	Fumaric acid	19.8 ± 4.5	1.5 ± 0.4	28.6 ± 4.0	−12.5	1.4	19.1
	Glucaric acid	1.2 ± 0.3	2.7 ± 0.4	3.0 ± 0.1	2.3	2.6	1.1
	Gluconic acid	0.6 ± 0.1	11.8 ± 2.2	5.8 ± 2.1	20	10	−2
	Glyceric acid	7.0 ± 0.9	6.9 ± 1.3	3.7 ± 0.2		−2	−1.9
	Glycolic acid	5.2 ± 0.6	5.1 ± 0.9	20.2 ± 1.8		3.9	4
	Lactic acid	358.9 ± 52.4	320.6 ± 36.2	682.9 ± 60.6	−1.1	1.9	2.1
	Malic acid	3.4 ± 0.6	ND	12.9 ± 2.3	<−100	3.7	>100
	Monomethylphosphate	104.8 ± 24.0	161.3 ± 2.0	258.1 ± 17.9	1.5	2.5	1.6
	Oxyphosphinyloxyacetate	3.8 ± 0.1	3.4 ± 0.3	6.6 ± 1.0	−1.1	1.7	1.9
	Pantothenate	ND	ND	2.2 ± 0.0		>100	>100
	Phenylpyruvic acid	ND	ND	3.4 ± 0.4		>100	>100
	Pyruvic acid	58.1 ± 10.5	38.9 ± 15.8	31.4 ± 4.8	−1.4	−2	−1.2
	Succinic acid	36.1 ± 8.7	31.6 ± 8.2	42.9 ± 9.6	−1.1	1.2	1.4
	α-Glycerophosphate	824.3 ± 131.3	1487.9 ± 121.8	920.7 ± 76.9	1.8	1.1	−1.6
	α-Ketoglutaric acid	30.7 ± 6.9	11.1 ± 2.1	18.2 ± 2.7	−2.8	−1.7	1.6
	β-Lactate	3.6 ± 0.8	2.0 ± 0.2	8.5 ± 1.7	−1.7	2.3	4.3
	β-Phenyllactic acid	3.0 ± 0.6	1.7 ± 0.2	7.3 ± 0.7	−1.7	2.4	4.3
**Sugars**	Arabitol	6.3 ± 1.3	12.0 ± 2.3	4.8 ± 0.7	1.9	−1.3	−2.5
	Fructose	40.5 ± 7.5	4.0 ± 0.8	1.8 ± 0.2	−10	−23	−2.2
	Fructose-6-P	6.7 ± 2.0	5.2 ± 1.0	3.0 ± 0.7	−1.3	−2.5	−1.7
	Galactitol	ND	2.1 ± 0.2	2.0 ± 0.3	>100	>100	−1.1
	Galactopyranose	2.2 ± 0.5	3.4 ± 0.7	2.0 ± 0.1	1.5	−1.1	−1.7
	Galactose	5.1 ± 1.3	7.9 ± 0.8	1.5 ± 0.3	1.5	−3.3	−5.3
	Glucose-1-P	20.3 ± 5.2	13.8 ± 3.4	2.3 ± 0.6	−1.4	−10	−6
	Glucose-6-P	13.6 ± 0.7	1.6 ± 0.3	0.5 ± 0.1	−8.5	−27.2	−3.2
	Glycerol	569.8 ± 71.4	668.7 ± 76.0	611.0 ± 103.9	1.2	1.1	−1.1
	Glycerol-2-P	28.2 ± 7.4	12.1 ± 3.0	7.6 ± 1.5	−2.5	−3.3	−1.6
	Inositol	3.1 ± 1.0	12.8 ± 1.1	0.1 ± 0.0	4.1	−31	−128
	Inositol, -chiro-	0.8 ± 0.1	1.5 ± 0.3	1.9 ± 0.1	1.8	2.2	1.3
	Mannitol	178.1 ± 15.6	29.5 ± 7.2	32.3 ± 3.1	−6	−5.5	1.1
	Mannitol-P	36.6 ± 3.2	9.0 ± 1.3	14.3 ± 2.8	−4.1	−2.6	1.6
	Mannose	7.2 ± 1.0	12.8 ± 2.4	1.2 ± 0.2	1.8	−6	−10.7
	Mannose-6-P	13.2 ± 2.9	ND	ND	<−100	<−100	
	Ribitol	122.9 ± 22.0	24.2 ± 4.4	76.2 ± 15.4	−5	−1.6	3.1
	Ribose	47.9 ± 9.4	42.0 ± 7.1	25.0 ± 3.3	−1.1	−2	−1.7
	Ribose-5-P	4.3 ± 0.5	30.6 ± 1.8	12.5 ± 2.0	7.2	2.9	−2.4
	Sedoheptulose	2.3 ± 0.7	2.2 ± 0.0	ND		<−100	<−100
	Sedoheptulose-7-P	1.0 ± 0.0	ND	ND	<−100	<−100	
	Sucrose	14.5 ± 2.5	1.4 ± 0.7	6.4 ± 0.2	−10	−2.3	4.6
	Trehalose	2.5 ± 0.3	17.6 ± 0.7	3.4 ± 0.8	7	1.4	−5.2

**Figure 2:**
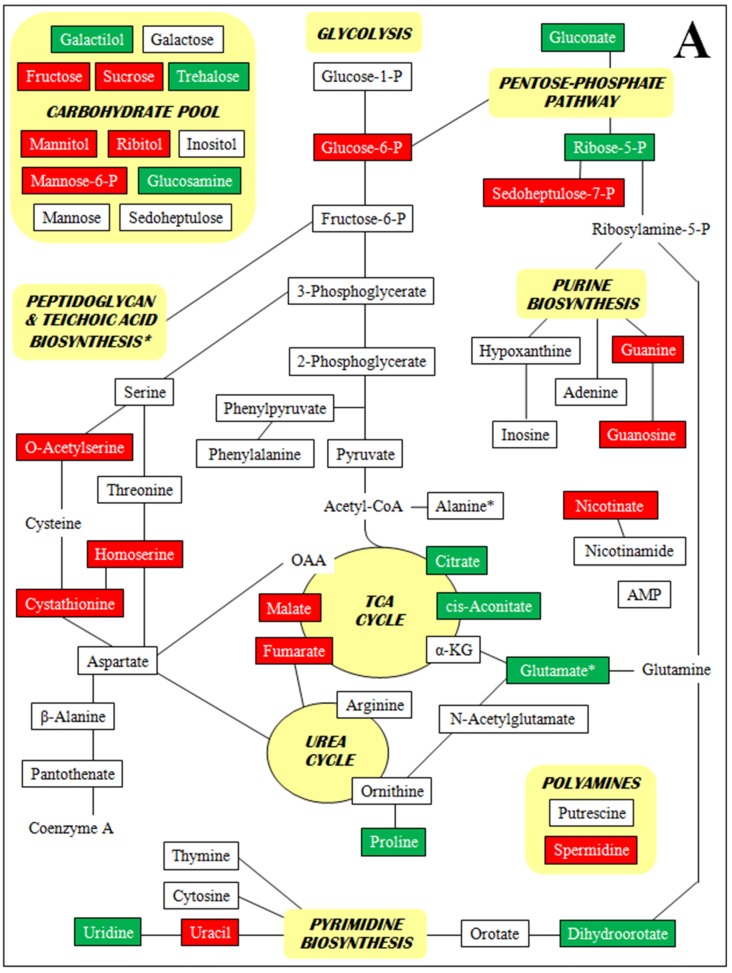

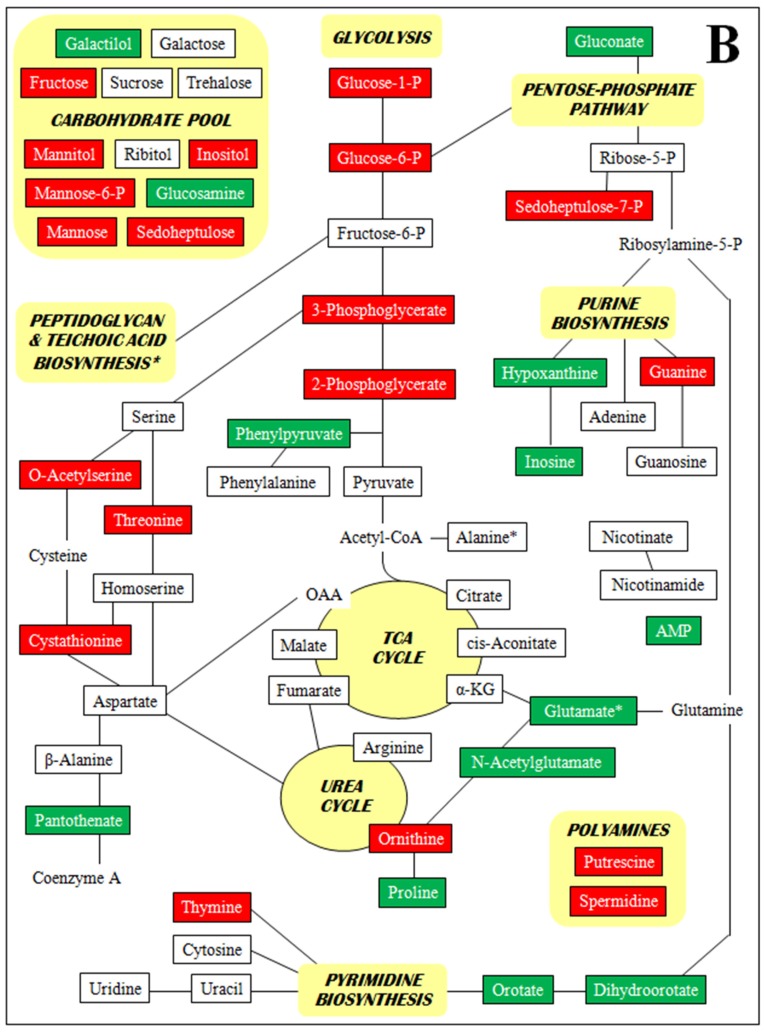

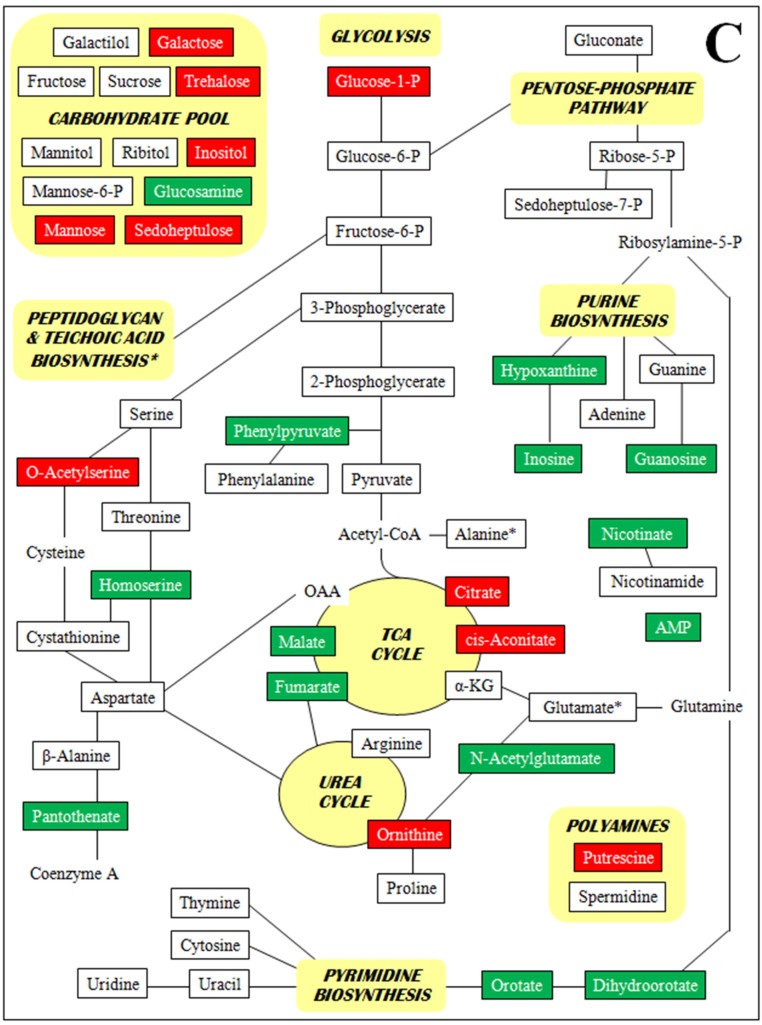
Metabolites with at least one five-fold relative concentration difference between (**A**) VISA 13136p^−^m^+^V5 relative to VSSA 13136p^−^m^+^, (**B**) VISA 13136p^−^m^+^V20 relative to VSSA 13136p^−^m^+^ and (**C**) VISA 13136p^−^m^+^V20 relative to VISA 13136p^−^m^+^V5, as well as their positions in metabolic pathways. Bordered terms: metabolites identified by metabolomic analyses; green shading: five-fold or greater relative concentration increases; red shading: five-fold or greater relative concentration decreases.

Figure 2 is a set of diagrams of central intermediary metabolic pathways with detected metabolites indicated along with increases and decreases in concentration for all three comparisons of relative concentrations (each VISA *versus* the parent VSSA and VISA 13136p^−^m^+^V20 *vs.* VSSA 3136p^−^m^+^V5). For clarity, only metabolite relative concentration changes of five-fold or greater are presented. Forty five of the 98 metabolites identified had at least one relative concentration change of five-fold or greater among the three strain comparisons. By metabolite class, these were: 13 amines and polyamines, eight amino acids, nine polar organic acids and 15 sugars. These 45 metabolites, their relative concentrations per strain, and relative concentration fold-changes for all three strain comparisons are listed in Supplementary Table S4. The Figure 2 diagrams are not intended to be complete pathways, and many metabolites are involved in numerous metabolic processes; and for some, such as polyamines and unphosphorylated sugars, there is uncertainty around their metabolic roles. The pathways presented represent those that seem relevant given the context of the other metabolites identified and the results of the genomic and transcriptomic analyses.

A number of metabolites were only detected in one or two of the three strains employed in this study. Particularly striking were the absence of guanosine from 13136p^−^m^+^V5 and the accumulation of AMP, *N*-acetylglutamic acid, pantothenate and phenylpyruvic acid in 13136p^−^m^+^V20. Polyamine relative concentrations were sequentially diminished as greater vancomycin tolerance accrued over the course of VISA selection. Initially, the spermidine relative concentration was reduced 9.4-fold by the selection of 13136p^−^m^+^V5 from VSSA 13136p^−^m^+^, while putrescine’s was unchanged; then the selection of 13136p^−^m^+^V20 from 13136p^−^m^+^V5 led to a 5.4-fold reduction in putrescine that was accompanied by a further, small reduction in spermidine. Several sugars were sequentially diminished in a similar fashion, notably glucose-1-phosphate, glucose-6-phosphate, and fructose.

The amino acids consistently present in the highest concentrations were alanine, aspartic acid, glutamic acid, leucine, lysine, proline and pyroglutamic acid. In general, the concentrations of these amino acids were increased in strains 13136p^−^m^+^V5 and 13136p^−^m^+^V20 compared to the parent strain. Aspartic acid was increased 2.3- and 2.4-fold, respectively, in the two VISA strains, glutamic acid 14- and 15-fold, leucine 2.8- and 3.8-fold and proline 14- and 22-fold.

There were high concentrations at a similar level in all three strains of pyroglutamate, which is the cyclic lactam of glutamic acid [60]. Although pyroglutamic acid is almost ubiquitously present in cells from Archaea to humans, it is a neglected metabolite. Pyroglutamate is formed by a number of different routes, including from the degradation of glutathione, spontaneous cyclization of glutamate phosphate and non-enzymatically from glutamate and glutamine. We are unaware of previous reports of its occurrence in *S. aureus*.

Glycerol was present at similar high levels in all three strains. Alpha glycerophosphate was present in all three strains. Mannitol relative concentration was significantly decreased in the VISA strains. Acid production from mannitol is a species characteristic of *S. aureus*. Lactic acid was present at high levels in all three strains.

## 3. Discussion

**Figure 3:**
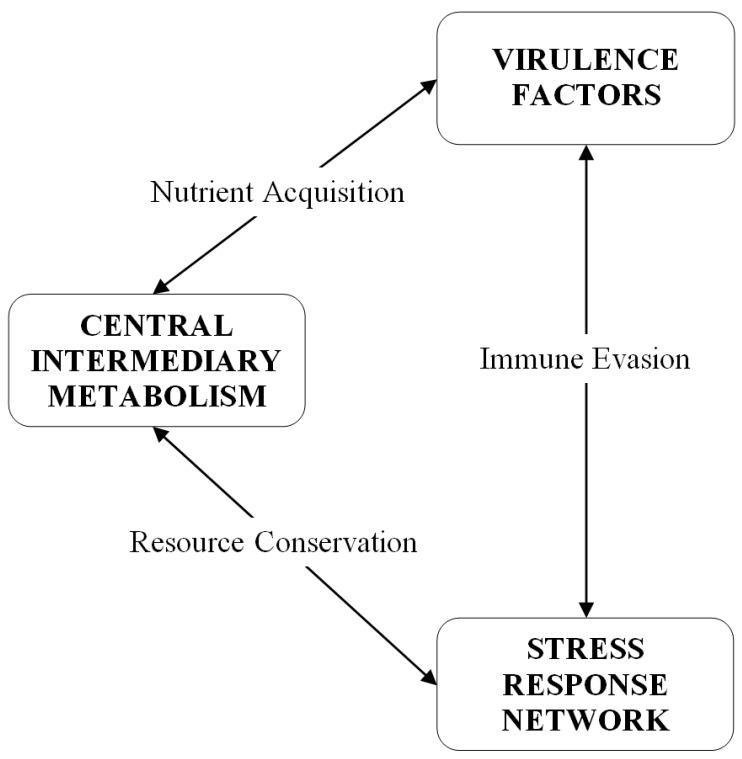
Conceptual diagram of the relationship between central intermediary metabolism, virulence factors, and the stress response network. Virulence is related to central intermediary metabolism through the production of soluble exoprotein virulence factors, regulated in a cell density-dependent fashion, which destroy host tissue in order to make nutrients available to a growing bacterial population. Cell-associated virulence factors are primarily defenses against host immune responses, working in conjunction with stress responses to immune system oxidative insults and damage sensing and responses to cell wall-active antibiotics. The stringent response is a stress response that coordinates a downregulation of central intermediary metabolism, and protein and nucleic acid synthesis, in order to conserve resources during periods of nutritional scarcity.

It has become apparent that the stress response network and virulence are intrinsically interwoven with central intermediary metabolism. Figure 3 is a diagram of the relationships between these three aspects of bacterial physiology. Secreted virulence factors, such as proteases and hemolysins, destroy host tissue in order to generate nutrients for growing pathogen populations. The released nutrients are transported into the *S. aureus* cell to serve as substrates for central intermediary metabolic pathways. Metabolic pathways are modulated to minimize damage from host immune response oxidative and nitrosative assaults [61]. Cell surface associated virulence factors, such as adhesions, promote host tissue colonization and aid in the evasion of host immune responses that are triggered by the activities of the secreted proteins. The production of virulence factors in *S. aureus* is density-dependent and regulated by a complicated network of global regulatory loci, but also by stress-related adjustments of central metabolic pathways, including the tricarboxylic acid (TCA) cycle. Some TCA cycle enzymes are upregulated in biofilm-associated cells *versus* planktonic culture [62,63]. Many of these global regulators, such as alternative sigma factor σ^B^ and members of the staphylococcal accessory regulator (Sar) family, exert regulatory influence over not only virulence factors, but the *S. aureus* general stress response and expression of antimicrobial resistances, including vancomycin intermediate susceptibility [64].

The alarmone-mediated nutritional stress response, known as the stringent response, has been linked to activated stress responses and methicillin resistance [45]. The *S. aureus* starvation response involves expression changes to genes associated with DNA repair and oxidative stress resistance [65]. *rpoB* mutations associated with conversion from VSSA to VISA also impart characteristics of the stringent response, reduced expression of genes encoding ribosomal proteins and upregulation of genes encoding amino acid biosynthesis enzymes, in addition to a slowed growth rate [66]. RNA polymerase subunit encoding genes *rpoB* and *rpoC* were downregulated 3.1- and 2.1-fold in 13136p^−^m^+^V20, respectively, but expression was unchanged in 13136p^−^m^+^V5. The stringent response is activated in MRSA in response to β-lactam antibiotics and leads to intermediary metabolic pathways optimized for energy production [67,68]. Stringent response induction by mupirocin in a heterogeneous MRSA dramatically increased the production of PBP2A and resulted in homogenous methicillin resistance expression [69]. Additionally, the link between virulence and the stringent response has been described for *S. aureus* and other bacteria [70]. Despite a dramatic upregulation of the *mecA* gene in VISA 13136p^−^m^+^V5 (48.5-fold *vs.* parent VSSA or 13136p^−^m^+^ V20), the oxacillin MIC of 13136p^−^m^+^V5 was comparable to those of the other two strains. Expression of two other genes encoding penicillin-binding proteins changed only slightly, *pbp2* down 2.3-fold in 13136p^−^m^+^V20 and *pbp3* down 2.5-fold in13136p^−^m^+^V5, despite the reported importance of PBP2 to laboratory-selected VISA [71].

Multiple studies have identified genes associated with decreased susceptibility to vancomycin. These studies have been summarized by Howden *et al.* [3,4], who have attempted to make some generalizations. Mutations in the regulatory genes, *walKR*, *vraRS* and *graRS*, and the gene encoding RNA polymerase subunit (*rpoB*) have been most frequently associated with the VISA phenotype. WalKR is an essential two-component system involved in the control of cell wall metabolism, particularly in controlling the expression of peptidoglycan hydrolases [72]. VraSR controls the cell wall stress stimulon [5,73]. GraSR is a two-component system associated with vancomycin resistance [74] and more generally with sensing antimicrobial peptides [75]. We did not detect mutations in any of these genes in our VISA strains. However, mutations were detected in *stp1*, *fmtB* and *tarO*, which are all involved in cell wall metabolism. Matsuo *et al.* [76] have identified mutations associated with the transition from hVISA to VISA. These authors have suggested that mutations in genes involved in protein synthesis, such as translation elongation factor G and translation initiation factor IF-1, in our case, may reduce protein synthesis and allow more use of amino acids for peptidoglycan synthesis. Indeed, the levels of several peptidoglycan precursor amino acids were elevated in our VISA strains.

Many of the mutations we encountered in our VISA strains have either not been or have only been rarely associated with the VISA phenotype. Five of the 12 13136p^−^m^+^ VISA mutations are in genes encoding products that interact with nucleic acids in protein synthesis or are associated with DNA damage/SOS responses: *comGB*, *fusA*, *infA*, *ssb1* and *dinG*. Often, a single stress will activate components from multiple stress responses. Oxidative stress and heat shock are known to elicit very broad stress responses [25]. However, SOS responses, such as recombination repair and competence mechanisms, are not commonly listed among the stress response alterations associated with the VISA phenotype.

That seven of the nineteen mutant loci identified in this study were in L54a prophage gene *ssb1* is intriguing. Single-stranded DNA binding proteins of L54a and similar prophages are thought to function as positive transcriptional regulators of integrase and perhaps other recombination-related phage genes [28,77]. Prophage genomes are known to harbor bacterial virulence determinants that can be regulated by global regulatory elements, such as the *agr* system [78], and cases have been reported of phage proteins exerting regulatory influence over host gene expression [79,80]. Cell wall biosynthesis disruption in MRSA COL results in downregulation of 33 L54a genes and upregulation of only two (none were *ssb1*), interpreted as a reinforcement of the lysogenic state, because stress is known to activate the SOS system and thereby the entry of the prophage into the lytic cycle [27]. The fact that mutations were identified in three L54a loci in VISA 13136p^−^m^+^V5 and additional mutations accumulated during the selection of increased resistance in VISA 13136p^−^m^+^V20, and that there were no mutations in other L54a genes such as those encoding the excisionase, suggests that *ssb1* may have a regulatory function that was the target of VISA selection. It seems unlikely that so many mutations in one gene would be necessary to simply disable lytic cycle capabilities. Likewise, mutations in *fusA*, *infA* and *stp1* may serve to modulate regulatory functions. Competence damage-inducible protein CinA, with functional similarities to both the *comGB* and *dinG* gene products, was identified as a member of the cell wall stress stimulon by Utaida *et al.* [5]. Interestingly, in relation to *comGB*, L54a itself cannot induce competence as a prophage [81].

Matsuo *et al.* [66] described the importance of regulation to the VISA resistance mechanism. The greater ratio of up- to down-regulated regulatory function genes for VISA 13136p^−^m^+^V20 (6:11) than 13136p^−^m^+^V5 (4:15) supports this observation. This is also true for the *stp1* SNP reversion when VISA 13136p^−^m^+^V20 was selected from VISA 13136p^−^m^+^V5. Several regulatory genes showed modest downregulation in 13136p^−^m^+^V5: *arlSR*, *glnR*, *pyrR*, *rsbU*, *agrA*, *agrB*, *agrC2* (but not *agrD*), *icaR*, *sarA*, *sarV*, *rpoF* (encodes stress response RNA polymerase sigma factor σ^B^) and *rsbV* (encodes an anti-anti-sigma factor). However, *saeS*, *sarR* and *vraR* showed modest upregulation in 13136p^−^m^+^V5, and the downregulation of both *rpoF* and *rsbV* together was unusual in that this would have opposite effects relative to σ^B^-mediated regulation. In 13136p^−^m^+^V20, *sarA* homologs *sarS* and *rot* (repressor of toxins) were highly upregulated at 8.9- and 12-fold *versus* parent VSSA, respectively. *agrA*, *agrB*, *agrC2* and *agrD* genes, as well as *saeS*, *sarA* and *rsbW* were downregulated in 13136p^−^m^+^V20, while *arlSR*, *glnR*, *pyrR*, *rpoF*, *rsbV*, *sarR*, *sarV* and *vraR* had no expression change *versus* the progenitor 13136p^−^m^+^. *saeS* is the sensor histidine kinase gene of a two-component regulation system that is upregulated by β-lactams and vancomycin in some other strains [82], and *sarA* is important for intrinsic antimicrobial resistance [83]. The complexity of the network of regulatory elements that controls virulence factor expression also extends to capsules and biofilms. The general downregulation of capsular polysaccharide biosynthesis genes can be directly interpreted as capsule production inhibition, although typically *cap* genes have been found to be overexpressed in VISA strains [3,4]. Whether or not biofilm formation was favored or inhibited in the two VISA cannot be discerned from the pattern of expression changes among regulatory loci, although the general downregulation of the protease-encoding genes is favorable to biofilm formation [84].

Secreted and cell surface-associated virulence factor gene expression patterns were similarly complicated. *aur*, encoding the zinc metalloproteinase aureolysin, was overexpressed 8.2-fold in 13136p^−^m^+^V5. Aureolysin has been shown to cleave complement component C3 and is proposed to mediate immune evasion [85]. Gene *sspB* encodes a cysteine protease precursor and was upregulated 3.7-fold in 13136p^−^m^+^V5, but unchanged in 13136p^−^m^+^V20. This gene was reported as downregulated in another VISA strain, JH9 [6]. Genes *sspC* and *sspA*, encoding a cysteine protease and V8 serine protease, respectively, were downregulated in both 13136p^−^m^+^V5 and 13136p^−^m^+^V20. Ingavale *et al.* [86] suggested that decreased levels of SspA may affect the processing of autolysins, such as Atl, resulting in altered autolytic activity, a characteristic displayed by our VISA strains. Serine protease loss may also affect other proteases, specifically cysteine protease proteolytic maturation [87]. The *spa* gene, encoding protein A, had no expression change in 13136p^−^m^+^V5 but surprisingly was upregulated 11.5-fold in 13136p^−^m^+^V20. Downregulation of this gene is the typical observation in VISA strains [3,4].

All 16 SNPs in VISA 13136p^−^m^+^V20 were in genes with known associations to stress responses, primarily to cell wall antibiotics and DNA damage. The *tarO* gene product is a membrane-associated protein that catalyzes the initial step in the biosynthesis of cell wall teichoic acid, a polymer of ribitol phosphate subunits [13]. The functions of teichoic acid are not fully understood, but it has roles in VISA characteristics such as cell wall structure, cell separation, autolysis and β-lactam susceptibility. Teichoic acid D-alanylation, mediated by *dlt* gene products, promotes biofilm formation and cell adhesion and is associated with the regulation of autolysis and virulence [88]. Both *dlt* genes were downregulated three-fold, but only in VISA 13136p^−^m^+^V5. Only VISA 13136p^−^m^+^V5 had the mutation in *tarO*, and it had the largest decrease in the metabolite ribitol relative concentration (five-fold, *versus* only 1.6-fold in VISA 13136p^−^m^+^V20). VISA 13136p^−^m^+^V20 has only 70% of the cell wall phosphorus of the parent VSSA, whereas VISA 13136p^−^m^+^V5 has 92% of the parent level [13]. Reductions in ribitol concentration did not correlate with lower phosphorus levels in VISA purified cell walls, which is indicative of lower teichoic acid content.

Fructose levels were 10- and 23-fold lower in 13136p^−^m^+^V5 and 13136p^−^m^+^V20, respectively, *versus* the relative concentration in the parent VSSA. Fructose phosphate levels were slightly lower in the two VISA than the parent. Expression of locus SAV0698, encoding a putative fructose utilization (*fru*) operon repressor, was upregulated 2.3- and 4.3-fold in 13136p^−^m^+^V5 and 13136p^−^m^+^V20, respectively. Factors contributing to the diminished fructose pool could be the repression of genes encoding enzymes required for uptake and utilization or the diversion of available fructose toward upregulated peptidoglycan synthesis. There are problems with both of these hypotheses, however. The uptake mechanism encoded by the *fru* operon is a phosphotransferase system, but it was unphosphorylated fructose whose relative concentrations changed dramatically. Furthermore, the thickened cell walls characteristic of the VISA phenotype are generally only produced in the presence of glycopeptides. Analyses in this study were performed on cells grown in antibiotic-free medium. Additionally, in 13136p^−^m^+^V5, the *glmS* gene is downregulated, but not in 13136p^−^m^+^V20. GlmS catalyzes the initial step in UDP-*N*-acetylglucosamine biosynthesis, in which fructose as fructose-6-phosphate is incorporated into the cell wall biosynthetic pathway.

Components of the *S. aureus* general stress response network were selectively deployed in the two VISA, as three of the eight genes whose expression was highly downregulated (≥8-fold *versus* parent VSSA) were associated with stress responses. These were *asp23*, encoding alkaline shock protein 23, locus SAV2185, encoding a putative transporter of osmoprotectant glycine betaine, and SACOL1114, encoding a putative Mn^2+^/Fe^2+^ transporter associated with oxidative stress management. The remaining five genes encoded two enterotoxins (*sec*, *seb*), a pseudogene with homology to integrase/recombinase proteins and two hypothetical proteins. No genes were upregulated ≥8-fold in both VISA.

There was evidence of stress response activation or modification in metabolomic data. The *ica* operon was strongly upregulated in VISA 13136p^−^m^+^V5, but not VISA 13136p^−^m^+^V20. Production of a number of virulence factors, including the intercellular adhesin proteins encoded by the *ica* operon, are regulated by TCA cycle changes in response to stress. An increase in ribose concentration has been linked to TCA cycle stress and an accompanying pentose phosphate pathway regulation modulation [63]. Ribose-5-phosphate concentration was 7.2-fold greater in VISA 13136p^−^m^+^V5 than the parent VSSA, although only up 2.9-fold in VISA 13136p^−^m^+^V20, while concentrations of ribose itself were comparable in VISA 13136p^−^m^+^V5 and the parent VSSA but two-fold lower in VISA 13136p^−^m^+^V20 than the parent strain.

Alcohol dehydrogenase is a fermentation pathway enzyme encoded by the *adh1* gene, whose expression was decreased 7.8-fold in VISA 13136p^−^m^+^V5 *versus* the parent VSSA, representing a reduced potential for fermentation, while expression was increased 4.7-fold in VISA 13136p^−^m^+^V5 *versus* the parent VSSA, representing an increased potential for fermentation. The *pdhC* gene encodes a component of the pyruvate dehydrogenase complex, which converts pyruvate to acetate. Expression of *pdhC versus* the parent 13136p^−^m^+^ was unchanged in 13136p^−^m^+^V5 and downregulated 3.1-fold in 13136p^−^m^+^V20, indicating that fermentation to acetate was not occurring. A shift away from anaerobic growth is consistent with the fact that acetate was not detected by the metabolomic analysis, pyruvic acid levels declined only slightly in the two VISA in comparison to the VSSA parent, and nitrate extrusion protein gene *narK* was downregulated in 13136p^−^m^+^V5.

Adenosine monophosphate (AMP) was not detected in 13136p^−^m^+^ or 13136p^−^m^+^V5, but was at detectable levels in 13136p^−^m^+^V20. In *S. aureus*, the PurA protein is a substrate for phosphorylation by Stk1, the cognate serine threonine phosphatase to the Stp1 serine threonine kinase encoded by the *stp1* gene [20] containing an SNP in 13136p^−^m^+^V5 that reverted during selection of 13136p^−^m^+^V20. The *purA* gene encodes adenylosuccinate synthase, which catalyzes the final step in AMP biosynthesis. However, *purA* was downregulated 2.4-fold in 13136p^−^m^+^V20, but unchanged in 13136p^−^m^+^V5, so the mutant Stp1 protein was not responsible for the increased AMP level in 13136p^−^m^+^V20. Therefore, the accumulation of AMP and downregulation of a gene encoding its biosynthesis may be due to a reduced demand for ATP in 13136p^−^m^+^V20. Energy conservation is a stringent response characteristic, as is the general downregulation of purine and pyrimidine metabolism genes observed in both VISA.

The major proline biosynthetic pathway in *S. aureus* is through arginine and ornithine via the urea cycle, not from glutamate [89]. Accumulation of osmoprotectants, including trehalose and especially proline, is an important osmotic stress countermeasure employed by *S. aureus*. Trehalose was present in 13136p^−^m^+^V5 and 13136p^−^m^+^V20 at relative concentrations seven- and 1.4-fold higher than in the parent strain. This is consistent with an activated stress response network in VISA, but not with the gene expression data for the trehalose-specific phosphotransferase system encoded by *treP* (downregulated 4.2-fold and upregulated 5.3-fold in 13136p^−^m^+^V5 and 13136p^−^m^+^V20, respectively). The TreP system is phosphoenolpyruvate (PEP) dependent, however, and PEP was not among the identified metabolites, suggesting that other uptake systems may have been utilized for trehalose and indicating that no significant gluconeogenesis was occurring in any of the three strains.

Proline relative concentrations were extremely elevated *versus* the parent VSSA in both 13136p^−^m^+^V5 (14-fold) and 13136p^−^m^+^V20 (22-fold). Citrate, *cis*-aconitate and glutamate, metabolites prior to the urea cycle branch point in the TCA cycle, all accumulated in 13136p^−^m^+^V5, while fumarate and malate concentrations, which are at or after the urea cycle branch point, were reduced. TCA cycle intermediate *cis*-aconitate was not detected in parent VSSA or 13136p^−^m^+^V20, only in 13136p^−^m^+^V5. In 13136p^−^m^+^V20, the identified TCA cycle intermediates, except *cis*-aconitate, were all within five-fold of parent strain relative concentrations, but many intermediates in glycolysis were reduced at least five-fold *versus* the parent. This suggests elevated flux through glycolysis to allow the TCA cycle to maintain such high proline levels. However, *S. aureus* also imports proline via the ProP low-affinity transport system in response to osmotic stress or a high-affinity PutP transport system in response to nutrient limitation [90]. Gene expression for locus SAV0573, encoding a ProP proline/betaine transporter homolog, was upregulated 3.7-fold in 13136p^−^m^+^V5 *versus* the parent strain, consistent with elevated proline concentrations and an activated stress response network. Data were not available for 13136p^−^m^+^V20 *proP* expression. Overall, the results suggest that 13136p^−^m^+^V5 was more reliant on transport into the cell via ProP to accumulate proline than 13136p^−^m^+^V20, which, in turn, relied more on proline biosynthesis from arginine via the urea cycle. Osmoprotectant uptake from the environment is a more rapid response to osmotic stress than *de novo* biosynthesis. However, that becomes irrelevant if proline biosynthesis is an aspect of the stress response network in VISA 13136p^−^m^+^V20 that is permanently activated to maintain high proline concentrations. Glutamate accumulation may have been due to reduced demand from nucleotide biosynthesis pathways.

Nelson *et al.* [17] have suggested that there is decreased flow through the TCA cycle in VISA strains and that this is associated with decreased aconitase activity; however, no expression changes were identified for any of the genes in the *cit* operon. Of seven pyrimidine ribonucleotide biosynthetic pathway genes with expression changes in this study, two were downregulated and five unchanged in 13136p^−^m^+^V5 and six downregulated and one unchanged in 13136p^−^m^+^V20. The relative concentrations of pyrimidines, as well as purines, did not correlate well with biosynthetic gene expression patterns. McAleese *et al.* [6] reported *pyr* gene downregulation in VISA, and Reiss *et al.* [45] reported *pur* gene downregulation in MRSA COL during a mupirocin-induced stringent response. Hypoxanthine and inosine were detected in 13136p^−^m^+^V20, but not in 13136p^−^m^+^V5 or the parent 13136p^−^m^+^. These are purine salvage pathway substrates either taken up from the growth medium, perhaps due to activated elements of the starvation response, or are degradation products from excess adenine and adenosine after downregulated purine biosynthesis [65]. Likewise, the accumulation of pyrimidine biosynthetic intermediates, dihydroorotate and orotate, was consistent with a downregulated pyrimidine biosynthetic pathway. Their accumulation was sequential, as dihydroorotate but not orotate relative concentrations were dramatically increased *versus* the parent VSSA in 13136p^−^m^+^V5 and were substantially elevated again in 13136p^−^m^+^V20. Orotate levels were comparable in parent and 13136p^−^m^+^V5, with substantial accumulation only in 13136p^−^m^+^V20.

There were significant decreases in the polyamines putrescine (strain 13136p^−^m^+^V20) and spermidine (both strains). Polyamines play important roles in many cellular processes [91,92]. Joshi *et al.* [93] found polyamines to exhibit a strong antimutagenic effect on several strains of *S. aureus*. Lower levels of polyamines in VISA strains may facilitate the development of the VISA phenotype by increasing the frequency of mutations. Within the cell polyamines are often complexed with nucleic acids, where they are thought to serve a protective function against stress and possibly participate in the regulation of protein synthesis and DNA-binding protein interactions. Polyamines protect *E. coli* against oxidative stress and are a component of the acid stress response. On the cell surface polyamines have been shown to influence biofilm formation in *Bacillus subtilis* and a number of Gram-negative pathogens. Spermidine biosynthesis exerts a regulatory influence on *Streptococcus pneumoniae* autolytic activity, possibly through cell wall structural interactions [93,94].

Polyamine functions are generally poorly understood [94]. A complication to the assessment of this study’s polyamine data is the unique hypersensitivity of *S. aureus* to these molecules. It is questionable whether *S. aureus* is capable of synthesizing these polyamines [93]. Adenine phosphoribosyltransferase, encoded by the *apt* gene mutated in 13136p^−^m^+^V20 but not 13136p^−^m^+^V5, is involved in recycling polyamine biosynthesis byproducts in *E. coli*. Furthermore, in at least some Gram-positives, including *S. aureus*, the *relA* gene encoding an alarmone biosynthesis activity that mediates the stringent response is downstream of *apt*. *Streptomyces coelicolor* *relA* is transcribed from transcriptional read-through of *apt*, linking regulation of the stringent response and that of purine metabolism [31,34,35].

Glutamic acid is known to have roles in the osmoregulatory and stress physiology of *S. aureus* [89,95]. Alanine and glutamic acid are precursors of the l- and d-alanine, d-glutamic acid, and d-isoglutamine residues found in peptidoglycan. Their presence in increased concentrations in the VISA strains might be related to modifications in peptidoglycan structure and cell wall thickening in VISA strains [7,13,96,97,98]. However, levels of lysine, which is also found in *S. aureus* peptidoglycan, were decreased in the VISA strains. Possibly also related to peptidoglycan synthesis, glucosamine was undetectable in the parent strain but was present at 6.9/10mg dry weight in 13136p^−^m^+^V5 and 129.4/10mg dry weight in 13136p^−^m^+^V20. Peptidoglycan biosynthesis has been shown to be activated in some VISA strains [16], with an increased flow of glucose into the cell wall [74]. The levels of glycolytic pathway intermediates generally decreased sequentially from parent to 13136p^−^m^+^V5 and again from 13136p^−^m^+^V5 to 13136p^−^m^+^V20, suggesting an increasing reliance on the high flux of glucose through glycolysis as susceptibility to vancomycin increases during selection.

Mannitol is suspected of playing a role in osmoregulation in *S. aureus* [99]. Relative concentrations *versus* parent VSSA of mannitol and mannitol-6-phosphate decreased in 13136p^−^m^+^V5 (six- and 4.1-fold, respectively) and 13136p^−^m^+^V20 (5.5- and 2.6-fold, respectively). In the parent 13136p^−^m^+^, there was almost five-times the relative concentration of the unphosphorylated mannitol as in its VISA derivatives. The lower concentrations in the VISA can be explained by downregulated *mtlA* expression, which encodes a mannitol phosphotransferase system component, by 2.9- and 4.5-fold in 13136p^−^m^+^V5 and 13136p^−^m^+^V20, respectively. One reason for the dramatic proline accumulation in the VISA might have been to compensate for the unavailability of mannitol for service in osmoprotection.

Ideally, it would be possible to reveal elements of the VISA resistance mechanism by working backwards from phenotypic characteristics through metabolomic, then transcriptomic, and finally genomic data. However, interpretation of the omics data from this study presents several challenges. In-depth knowledge of the function of the gene products of many of the genes with SNPs is lacking, profoundly so for some of them (e.g., *ssb1*, *dinG* and *stp1*). A large proportion of gene expression changes identified by transcriptome analysis encode products in the hypothetical protein, unclassified, and unknown function gene functional groups (27% overall, rising to 40% of the high fold-change subset). This is not a novel observation [45]. As mentioned previously, metabolite relative concentration changes do not always correlate with the expression changes of genes encoding their biosynthetic enzymes and regulatory elements. This may be due in part to the fact that gene expression data do not provide information on gene product activity, and many enzymes and regulatory proteins engage in interactions with other regulatory molecules in ways that are not evident to broad analyses. For example, the alarmone synthetase and hydrolase activities are combined in a single, bifunctional protein in Gram-positive bacteria [45], and gene expression data on *relA* up- or down-regulation give no guidance on which activities are active at the time of sample processing. Furthermore, the single gene expression change in the mobile and extrachromosomal element functions gene functional group identified by the transcriptional analysis does not reflect the dominant position of the L54a prophage mutations within the genomic analysis.

## 4. Experimental Section

### 4.1. Strains and Growth Conditions

*S. aureus* strains 13136p^−^m^+^, 13136p^−^m^+^V5 and 13136p^−^m^+^V20 were studied [7]. They were grown in Tryptic Soy Broth (Becton Dickinson and Company, Cockeysville, MD, USA) at 37 °C with shaking at 200 rpm. Mid-exponential phase cells (OD_600nm_ 0.5) were used for transcriptional profiling and metabolomics analysis.

### 4.2. Complete-Genome Comparisons

These were performed with an array-based service provided by Nimblegen Systems Inc. (Madison, WI, USA) [100,101]. The reference genome was strain 13136p^−^m^+^ to which the genomes of 13136p^−^m^+^V5 and 13136p^−^m^+^V20 were compared.

### 4.3. Transcriptional Profiling

This was carried out as described in previous publications from this laboratory [102,103]. *S. aureus* genome microarrays version 6.0, provided by the Pathogen Functional Genomics Resource Center of the National Institutes of Allergy and Infectious Diseases, were used.

### 4.4. Microarray Validation by Real-Time Reverse Transcription-PCR (RT-PCR)

This was carried out as described by Song *et al.* [103] on selected genes.

### 4.5. Metabolomic Analysis

This was carried out as described by Singh *et al.* [104]. Briefly, 500 mL of mid-exponential phase cells growing in a 2-liter flask with shaking (200 rpm) at 37 °C were harvested, washed and extracted with 40% (vol/vol) methanol. The extracts were analyzed by liquid chromatography/mass spectroscopy and gas chromatography/mass spectroscopy. Data from each chromatogram were normalized to an internal standard of 10 mg/mL hentriacontanoic acid to allow dry weight comparisons between samples. Relative concentrations were calculated as metabolite peak area divided by internal standard peak area. These analyses were carried out at the Roy J. Carver Biotechnology Center, University of Illinois at Urbana-Champaign, Urbana, IL, USA. Three separate batches of each strain were analyzed, and the data were combined.

## 5. Conclusions

This study added new members to the lists of mutations and transcriptomes that are associated with the VISA phenotype. Overall trends in VISA characteristics were consistent with previous studies, including a great variability in the mutations and gene expression patterns among VISA of different lineages. This variability has been noted previously by Howden *et al.* [3,4,105]. Those authors and Gardete *et al.* [106] also confirm that VISA transcriptomes are generally consistent with decreased virulence. Parent VSSA 13136p^−^m^+^ in this study had been laboratory-propagated for decades, although for much of that time it was in frozen storage, and its VISA derivatives were selected *in vitro* and have never been passaged through animals. The extensive modulation of virulence determinants in these and other *in vitro*-selected and -propagated VISA demonstrates that virulence and central intermediary metabolism are inextricably intertwined. Extended *in vitro* passage has been shown to change the expression pattern of virulence determinants, but it does not eliminate them [107,108].

The importance of activated elements of the stress response network was reinforced by the results from this study. Stress response modulation in the VISA phenotype was evident across all three omics analyses, from mutations and expression changes with genes known to respond to stresses or encode stress-related products, to modulation of stress-protection metabolites, such as proline and polyamines. It was surprising how many mutations were identified in the two VISA strains that were associated with stress related to DNA damage and the SOS response, especially so for the strain with the higher vancomycin MIC (13136p^−^m^+^V20). The pattern of downregulation of central metabolic processes related to protein synthesis and purine and pyrimidine biosynthetic pathways indicates a stringent response-like state for the two VISA, as has been reported for both MRSA and VISA [45,66]. Downregulation of these processes was not simply a component of the slowed growth rates of these VISA *versus* their VSSA parent, for while central metabolism in VISA 13136p^−^m^+^V20 was clearly less active than that in 13136p^−^m^+^V5, their doubling times are very similar [7].

The greatest value derived from the metabolomics analysis was in providing physiologic evidence supporting the association of stress response activation and stringent response characteristics with the VISA phenotype. This was evident in progressive changes in metabolite relative concentrations that paralleled the selection of greater vancomycin resistance between VSSA 13136p^−^m^+^ and 13136p^−^m^+^V5, and then between 13136p^−^m^+^V5 and 13136p^−^m^+^V20. Numbers of stress response-related mutations consistently changed in this fashion, as did broad expression patterns for genes associated with aspects of metabolism related to the stress response network and central intermediary metabolism. This pattern was also evident in metabolite levels of osmoprotectants, polyamines, and intermediates in nucleotide biosynthesis and central carbon and energy utilization pathways.

## Supplementary Tables and Figures

1. **Supplementary Table S1.** Two-fold or greater gene expression changes in VISA 13136p^−^m^+^V5 and 13136p^−^m^+^V20 *versus* parent VSSA 13136p^−^m^+^.
2. **Supplementary Table S2.** Expression patterns by gene functional group in VISA 13136p^−^m^+^V5 and 13136p^−^m^+^V20 as number of genes upregulated and downregulated at least two-fold relative to gene expression in VSSA 13136p^−^m^+^.
3. **Supplementary Table S3.** Concordance of expression patterns *vs.* parent VSSA 13136p^−^m^+^ between 13136p^−^m^+^V5 and 13136p^−^m^+^V20 for the 335 genes with expression data for both VISA.
4. **Supplementary Table S4.** The 45 metabolites with at least one five-fold change among comparisons between 13136p^−^m^+^, 13136p^−^m^+^V5 and 13136p^−^m^+^V20.
5. **Supplementary Figure S1.** Number of genes by functional group upregulated and downregulated at least eight-fold in VISA 13136p^−^m^+^V5 (**A**) and 13136p^−^m^+^V20 (**B**) relative to gene expression in VSSA parent 13136p^−^m^+^.
6. **Supplementary Figure S2.** Metabolomic profiles by metabolite class (**A**–**D**).
7. **Supplementary File S1.** Metabolomic analysis background information.
