# Supplementary material for: Genomic, Transcriptomic and Metabolomic Studies of Two Well-Characterized, Laboratory-Derived Vancomycin-Intermediate Staphylococcus aureus Strains Derived from the Same Parent Strain
Source: Antibiotics (Basel). 2015 Feb 4;4(1):76–112. doi: 10.3390/antibiotics4010076 (PMC4790321; doi:10.3390/antibiotics4010076)
Supplement: Supplementary file 1 [file antibiotics-04-00076-s001.pdf]

Supplementary Material

Supplementary Table S1. Two-fold or greater gene expression changes in VISA 13136p^-m^+V5 and 13136p^-m^+V20 *vs*. parent VSSA 13136p^-m^+.

Functional Group	Locus ID	Gene	Protein	13136p−m+V5	13136p−m+V20
Amino Acid Biosynthesis	SAV2207	*alsS*	alpha-acetolactate synthase	4	3.2
	SAV1310	*glnA*	glutamine-ammonia ligase	−2.6	−2.1
	SACOL2105	*glyA*	serine hydroxymethyltransferase	−2.3	−4.2
	SAV2061	*ilvA*	threonine dehydratase	−2.5	
	SACOL2045	*ilvC*	ketol-acid reductoisomerase	−5.5	−6
	SACOL2042	*ilvD*	dihydroxy-acid dehydratase	−2.3	−3.8
	SAV2057	*leuA*	2-isopropylmalate synthase	−3.6	−4
	SAV2059	*leuC*	isopropylmalate isomerase large subunit		−3.3
	SAV2158	*mtlA*	PTS system, mannitol specific IIA component	−2.9	−4.5
	SAS2563	NA	putative histidinol dehydrogenase	−2.7	−2.1
	SACOL1773	*serA*	D-3-phosphoglycerate dehydrogenase	−2.6	−2.6
	SAV0460	*yrhB*	cystathionine gamma-synthase homolog		−2.5
Biosynthesis of Cofactors, Prosthetic Groups & Carriers	SAV2182	*asp23*	alkaline shock protein 23	−9.8	−19.7
	SACOL2428	*bioD*	dethiobiotin synthase		8.7
	SACOL0172	*entB*	Isochorismatase	−2.3	
	SAV2346	*fni*	isopentenyl pyrophosphate isomerase	2.2	2.2
	SACOL1719	*hemA*	glutamyl-tRNA reductase		2.2
	SACOL1715	*hemB*	delta-aminolevulinic acid dehydratase	2.2	

Table S1. *Cont.*

Functional Group	Locus ID	Gene	Protein	13136p−m+V5	13136p−m+V20
Biosynthesis of Cofactors, Prosthetic Groups & Carriers	SACOL1889	*hemE*	uroporphyrinogen decarboxylase		2.12
	SACOL1887	*hemG*	protoporphyrinogen oxidase		2.4
	SAV0924	*lipA*	lipoyl synthase	2.1	
	SACOL1049	*menA*	1,4-dihydroxy-2-naphthoate octaprenyltransferase	2.4	
	SAV2274	*moaC*	molybdenum cofactor biosynthesis protein C	2	2.1
	SACOL0774	NA	para-aminobenzoate synthase, component I	−6.7	−4.9
	SAS0284	NA	hypothetical protein	2.3	6.8
	SAS0678	NA	glutamine amidotransferase class-I protein	−8.6	−5.4
	SAV2472	NA	short chain dehydrogenase	−8.5	−5.2
	SAV2398	*nasF*	uroporphyrin-III C-methyl transferase	−4.9	No Data
	SAV1771	*ribD*	riboflavin specific deaminase	−2.1	−4.3
	SACOL1764	*thiI*	thiamine biosynthesis protein ThiI	2.6	
Cell Envelope	SACOL1062	*atl*	bifunctional autolysin		−2.4
	SAV2637	*aur*	zinc metalloproteinase aureolysin	8.2	
	SACOL0136	*cap5A*	capsular polysaccharide biosynthesis protein Cap5A	−6.7	−3.9
	SA0145	*capB*	capsular polysaccharide synthesis enzyme Cap5B	−5.4	
	23	*capJ*	capsular polysaccharide synthesis enzyme Cap5J		−2.2
	SAV0159	*capK*	capsular polysaccharide synthesis enzyme Cap5K		−2.8

Table S1. *Cont.*

Functional Group	Locus ID	Gene	Protein	13136p−m+V5	13136p−m+V20
Cell Envelope	SAV0932	*dltA*	D-alanine--D-alanyl carrier protein ligase	2.9	
	SACOL0936	*dltB*	DltB protein	3	
	SACOL1872	*epiE*	epidermin immunity protein F	−6	
	SACOL1871	*epiG*	epidermin immunity protein F	−11.2	
	SACOL1396	*fmtC*	FmtC protein	2.9	
	SACOL2003	*hlb*	integrated prophage inactivating a beta-hemolysin gene	3.1	No Data
	SAV2133	*hmrA*	HmrA	−4.3	
	SACOL2689	*icaA*	intercellular adhesion protein A	25.2	
	SACOL2692	*icaC*	intercellular adhesion protein C	4.6	
	SAV2667	*icaD*	intercellular adhesion protein D	15.9	
	SACOL2660	*isaB*	immunodominant antigen B		3.4
	SAV0041	*mecA*	penicillin binding protein 2 prime	48.5	
	SAV2099	*murA*	UDP-N-acetylglucosamine 1-carboxyvinyltransferase	−2.3	
	SAV1418	*murG*	N-acetylglucosaminyl transferase	−2.5	
	SAV2124	*murZ*	UDP-N-acetylglucosamine 1-carboxyvinyltransferase	2.2	
	SACOL0872	NA	OsmC/Ohr family protein	−8.5	−5.7
	SACOL1578	NA	FtsK/SpoIIIE family protein	−2.5	−7.3
	SACOL2578	NA	glycosyl transferase, group 2 family protein	−7.2	−3.1

Table S1. *Cont.*

Functional Group	Locus ID	Gene	Protein	13136p−m+V5	13136p−m+V20
Cell Envelope	SAR0392	NA	hypothetical protein	−7.4	−9.7
	SAR2053	NA	hypothetical protein	−5	−3
	SAS0108	NA	putative surface anchored protein	−3.6	5.6
	SAS0236	NA	cell wall metabolism protein ScdA	−2	9.5
	SAS0351	NA	hypothetical protein	−5.9	−15.9
	SAS2104	NA	hypothetical protein	−7.9	−3.6
	SAS2345	NA	hypothetical protein	−5.5	−7.5
	SAS2532	NA	putative surface anchored protein	4	4
	SAS2584	NA	hypothetical protein	−5.9	−2.5
	SAV0134	NA	hypothetical protein	−2.5	4.5
	SAV0178	NA	similar to integral membrane protein LmrP	−4.4	−5
	SAV0179	NA	similar to surfactin synthetase	−5.8	−7.1
	SAV0726	NA	similar to multidrug resistance protein and enterotoxin type C3	−3.1	−6.2
	SAV1450	*pbp2*	penicillin-binding protein 2		−2.3
	SAV1552	*pbp3*	penicillin-binding protein 3	−2.5	
	SAR0136	*sasD*	putative surface anchored protein	−2.9	5.6
	SAR2725	*sasF*	putative surface anchored protein		2.6
	SACOL0907	*seb*	staphylococcal enterotoxin B	−17.2	−18.3

Table S1. *Cont.*

Functional Group	Locus ID	Gene	Protein	13136p−m+V5	13136p−m+V20
Cell Envelope	SAV2009	*sec3*	enterotoxin type C3	−24.4	−21.5
	SAV0111	*spa*	Immunoglobulin G binding protein A precursor		11.5
	SAV2299	*ssaA*	secretory antigen precursor SsaA homolog	4.2	
	SACOL1057	*sspA*	V8 Protease	−4.7	−3.5
	SACOL1970	*sspB2*	cysteine protease precursor SspB	3.7	
	SAV1046	*sspC*	cysteine protease	−3.4	−8.6
Cellular Processes	SACOL0452	*ahpC*	alkyl hydroperoxide reductase, C subunit		3.1
	SACOL0451	*ahpF*	alkyl hydroperoxide reductase, subunit F		2.3
	SACOL2409	*fmhA*	fmhA protein		2.3
	SAV0320	*geh*	glycerol ester hydrolase	2.7	12
	SACOL2641	*gpxA2*	glutathione peroxidase	3.2	
	SACOL0034	*mecR1*	methicillin-resistance MecR1 regulatory protein	38.5	
	SACOL2291	NA	staphyloxanthin biosynthesis protein	4	−2.2
	SACOL2418	NA	IgG-binding protein SBI	2.3	5.6
	SAS2042	NA	putative non-heme iron-containing ferritin	2.3	6.8
	SACOL1010	*relA1*	GTP pyrophosphokinase	2.3	
	SAR0135	*sodM*	superoxide dismutase		4

Table S1. *Cont.*

Functional Group	Locus ID	Gene	Protein	13136p−m+V5	13136p−m+V20
Central Intermediary Metabolism	SAV0605	*adh1*	alcohol dehydrogenase	−7.8	4.7
	SACOL2576	*crtN*	dehydrosqualene desaturase	−2.1	
	MW2444	*ddh*	2-hydroxyacid dehydrogenase	−3	
	SAR2242	*glmS*	D-fructose-6-phosphate aminotransferase	−2.2	
	SACOL0032	*maoC*	(R)-specific enoyl-CoA hydratase	23.7	
	SAV0591	*mvaD*	mevalonate diphosphate decarboxylase	−3.2	−2.6
	SAV0590	*mvaK1*	mevalonate kinase	−2.7	−3.1
	SAV0680	NA	similar to lysine decarboxylase family	−2.6	−6
	SAV1536	NA	glycine dehydrogenase subunit 1	−2.5	−3.4
	SAV2033	NA	similar to nitroreductase family protein	−2.2	2.7
	SAV2328	NA	Dehydrogenase	−9.9	−4.4
	SAV2580	NA	hypothetical protein	−4.4	−2
	SAV2388	*narK*	nitrite extrusion protein	−7.6	
	SAV2627	*phoB*	alkaline phosphatase III precursor		2.2
	SACOL2282	*ureC*	urease, alpha subunit		−2.3
	SACOL2284	*ureF*	urease accessory protein UreF		−2.3
DNA Metabolism	SAV0002	*dnaN*	DNA polymerase III subunit beta	2.1	
	SACOL0005	*gyrB*	DNA gyrase, B subunit.		−2.2
	SACOL0678	NA	integrase/recombinase, phage integrase family	−6.5	−2.3
	SACOL1573	NA	integrase/recombinase, core domain family	−10.6	−12.1

Table S1. *Cont.*

Functional Group	Locus ID	Gene	Protein	13136p−m+V5	13136p−m+V20
Fatty Acid & PhosphoLipid Metabolism	SACOL2482	*fabG2*	3-oxoacyl-(acyl carrier protein) reductase, point mutation - premature stop	−6.7	−4.6
	SACOL0987	*fabH*	3-oxoacyl-(acyl carrier protein)		−2.2
	SAV1011	*fabI*	enoyl-(acyl carrier protein) reductase	2.6	
Hypothetical Proteins	MW0035	NA	hypothetical protein	−2.2	−5.4
	SACOL0067	NA	hypothetical protein	12.6	5
	SACOL0268	NA	hypothetical protein	2	6.6
	SACOL0489	NA	hypothetical protein	2.2	2
	SACOL0490	NA	hypothetical protein	3	3.2
	SACOL0625	NA	hypothetical protein	11.2	2.5
	SACOL0673	NA	hypothetical protein	−3.5	−2.9
	SACOL0738	NA	hypothetical protein	−3.4	−2.9
	SACOL0742	NA	hypothetical protein	−3.8	−2.8
	SACOL0849	NA	hypothetical protein	4	2.6
	SACOL0850	NA	hypothetical protein	4.9	3.7
	SACOL0851	NA	hypothetical protein	4.2	2.2
	SACOL0866	NA	hypothetical protein	−12.9	−3.8
	SACOL0908	NA	hypothetical protein	−3.8	−8.8
	SACOL0911	NA	hypothetical protein	3.8	−3.9
	SACOL0912	NA	hypothetical protein	−5.4	−8.3

Table S1. *Cont.*

Functional Group	Locus ID	Gene	Protein	13136p−m+V5	13136p−m+V20
Hypothetical Proteins	SACOL1041	NA	hypothetical protein	−2.6	−5.9
	SACOL1044	NA	hypothetical protein	3.5	3.2
	SACOL1574	NA	hypothetical protein	−5.4	−8.6
	SACOL1679	NA	hypothetical protein	−7.3	−10.3
	SACOL1680	NA	hypothetical protein	−6.3	−8.4
	SACOL2013	NA	hypothetical protein	−2.3	−2.1
	SACOL2174	NA	hypothetical protein	−7.4	−20.2
	SACOL2175	NA	hypothetical protein	−6.8	−17.3
	SACOL2300	NA	hypothetical protein	−3.9	−2.1
	SACOL2379	NA	hypothetical protein	−3.5	−2.8
	SACOL2489	NA	hypothetical protein	−2.7	−3.1
	SACOL2491	NA	hypothetical protein	−4.8	5.1
	SACOL2547	NA	hypothetical protein	−9.5	4.2
	SACOL2557	NA	hypothetical protein	2.4	−2
	SACOL2595	NA	hypothetical protein	−4.8	−2.6
	SACOL2720	NA	hypothetical protein	−9.4	−7
	SAR0592	NA	hypothetical protein	−12.3	−15.8
	SAR2739	NA	hypothetical protein	−3.8	−2.8
	SAS1017	NA	hypothetical protein	−4.7	−2.1

Table S1. *Cont.*

Functional Group	Locus ID	Gene	Protein	13136p−m+V5	13136p−m+V20
Hypothetical Proteins	SAS1090	NA	hypothetical protein	2.1	3.6
	SAS1587	NA	hypothetical protein	7.3	2.3
	SAS2047	NA	hypothetical protein	−8.1	−3.9
	SAS2396a	NA	hypothetical protein	−8.3	−6.4
	SAS2490	NA	hypothetical protein	−7.8	−3
	SAV0278	NA	hypothetical protein	2.5	2.3
	SAV0280	NA	hypothetical protein	2.4	2.2
	SAV0716	NA	hypothetical protein	−2.6	−3.5
	SAV0769	NA	cell-division inhibitor	−2.4	−2.2
	SAV0818	NA	hypothetical protein	−3.9	−3.7
	SAV0823	NA	hypothetical protein	−11.3	−3.1
	SAV1548	NA	hypothetical protein	−2.4	−2.3
	SAV2556	NA	hypothetical protein	12.8	3.6
	SAV2646	NA	hypothetical protein	6.2	4.5
	SAV2693	NA	hypothetical protein	−4.9	−3.3
Mobile & Extrachromosomal Element Functions	SACOL0134	NA	transposase, IS200 family, degenerate - contains one or more premature stops and/or frameshifts		2.4
Protein Fate	SAV1254	*clpY*	ATP-dependent protease ATP-binding subunit		−2.4
	SACOL1637	*dnaK*	dnaK protein		−2

Table S1. *Cont.*

Functional Group	Locus ID	Gene	Protein	13136p−m+V5	13136p−m+V20
Protein Fate	SAS0824	NA	putative cyclophilin type peptidyl-prolyl cis-trans isomerase	3.1	2.2
	SAS0983	NA	cysteine protease precursor	−3.8	−3.5
	SAV0372	NA	predicted PepSY family membrane peptidase propeptide	−4.1	−10.5
	SAV0753	*secA*	translocase		−2.6
Protein Synthesis	SACOL1961	*gatA*	glutamyl-tRNA(Gln) amidotransferase, A subunit		−2.6
	SACOL0574	*gltX*	glutamyl-tRNA synthetase		−2.2
	SACOL1622	*glyS*	glycyl-tRNA synthetase	2.2	
	SACOL1206	*ileS*	isoleucyl-tRNA synthetase	−2.1	−2.7
	SAV0517	*lysS*	lysyl-tRNA synthetase	2.3	
	SACOL1323	*miaA*	tRNA delta(2)-isopentenylpyrophosphate transferase	−2.9	−2.7
	SACOL1803	NA	pseudouridine synthase, family 1	−3.9	−2.7
	SACOL2239	*rplC*	ribosomal protein L3		−2.6
	SACOL2238	*rplD*	50S ribosomal protein L4		−2.7
	SACOL2227	*rplE*	ribosomal protein L5		−2
	SAV2218	*rplM*	50S ribosomal protein L13	−2	
	SACOL2229	*rplN*	ribosomal protein L14		−2.3
	SACOL2220	*rplO*	ribosomal protein L15		−2.1
	SACOL2232	*rplP*	50S ribosomal protein L16		−2.6
	SACOL2223	*rplR*	ribosomal protein L18		−2.5

Table S1. *Cont.*

Functional Group	Locus ID	Gene	Protein	13136p−m+V5	13136p−m+V20
Protein Synthesis	SACOL2234	*rplV*	ribosomal protein L22		−2
	SACOL2216	*rpmJ*	ribosomal protein L36		−2.2
	SACOL2233	*rpsC*	30S ribosomal protein S3		−2.2
	SACOL2222	*rpsE*	ribosomal protein S5	−2.2	
	SACOL1370	*rpsN1*	30S ribosomal protein S14		2.3
	SAV2241	*rpsQ*	30S ribosomal protein S17		−3.6
	SACOL2235	*rpsS*	ribosomal protein S19		−3.7
	SACOL1632	*rpsU*	ribosomal protein S21	−3.3	
	SAV0009	*serS*	seryl-tRNA synthetase		2.1
	SAV1683	*thrS*	threonyl-tRNA synthetase 1	−2.2	
	SAV0996	*trpS*	tryptophanyl-tRNA synthetase	2	
	SACOL1778	*tyrS*	tyrosyl-tRNA synthetase	2.2	2.9
Purines, Pyrimidines, Nucleosides & Nucleotides	SACOL2218	*adk*	adenylate kinase		−2.5
	SACOL1518	*cmk*	cytidylate kinase	3.2	
	SACOL2130	*deoD2*	Purine nucleoside phosphorylase		2.5
	SAV0390	*guaB*	inositol-monophosphate dehydrogenase	−3.5	
	SAS1134	NA	aspartate carbamoyltransferase catalytic subunit	−2.1	−3
	SACOL2634	*nrdG*	anaerobic ribonucleoside-triphosphate reductase activating protein	−3.8	−5.6
	SAV1841	*prsA*	peptidyl-prolyl *cis*/*trans* isomerase	3.4	

Table S1. *Cont.*

Functional Group	Locus ID	Gene	Protein	13136p−m+V5	13136p−m+V20
Purines, Pyrimidines, Nucleosides & Nucleotides	SAV0017	*purA*	adenylosuccinate synthase		−2.4
	SACOL1075	*purC*	phosphoribosylaminoimidazole-succinocarboxamide synthase	−5.3	
	SAV1074	*purD*	phosphoribosylamine-glycine ligase	−9.5	−2.3
	SAV1070	*purF*	phosphoribosylpyrophosphate amidotransferase	−5.4	−2.9
	SACOL1082	*purH*	phosphoribosylaminoimidazolecarboxamide formyltransferase/IMP cyclohydrolase	−7.2	−2.9
	SAV1065	*purK*	phosphoribosylaminoimidazole carboxylase carbon dioxide-fixation chain PurK homolog	−2.5	−2.2
	SAV1069	*purL*	phosphoribosylformylglycinamidine synthetase	−6.2	−3.2
	SAV1071	*purM*	phosphoribosylaminoimidazole synthetase	−8.4	−2.2
	SAV1072	*purN*	phosphoribosylglycinamide formyltransferase	−9.5	−2.5
	SACOL1077	*purQ*	phosphoribosylformylglycinamidine synthase I	−5.3	
	SACOL1076	*purS*	phosphoribosylformylglycinamidine synthase, PurS protein	−4.8	−2.8
	SAV1202	*pyrAA*	carbamoyl-phosphate synthase small subunit	−3.5	−4.5
	SAV1203	*pyrAB*	carbamoyl-phosphate synthase large subunit		−2
	SAV1201	*pyrC*	Dihydroorotase	−2.6	
	SACOL2606	*pyrD*	dihydroorotate dehydrogenase		−2.1
	SACOL1217	*pyrE*	orotate phosphoribosyltransferase		−2.2
	SACOL1216	*pyrF*	orotidine 5'-phosphate decarboxylase		−3.6
	SACOL2119	*pyrG*	CTP synthetase		−2
	SAV1258	*smbA*	uridylate kinase	2.5	

Table S1. *Cont.*

Functional Group	Locus ID	Gene	Protein	13136p−m+V5	13136p−m+V20
Purines, Pyrimidines, Nucleosides & Nucleotides	SACOL1462	*thyA*	Thymidylate	−2.9	
	SAR1690	*udk*	uridine kinase	2.2	
Regulatory Functions	SACOL2026	*agrA*	accessory gene regulator protein A	−2.8	−9
	SACOL2023	*agrB*	accessory gene regulator protein B	−2.8	−5.7
	SACOL2025	*agrC2*	accessory gene regulator protein C	−2.1	−4.8
	SACOL2024	*agrD*	accessory gene regulator protein D		−5.1
	SACOL1328	*glnR*	glutamine synthetase repressor	−4	
	SACOL1324	*hfq*	hfq protein, putative	−2.8	−2.4
	SAV2665	*icaR*	*ica* operon transcriptional regulator	−2.4	
	SACOL0890	NA	transcriptional regulator, Cro/CI family	−3.1	−3.1
	SACOL2147	NA	transcriptional antiterminator, BglG family/DNA-binding protein	−12	−4.4
	SAV0698	NA	similar to transcription repressor of fructose operon	2.3	4.3
	SAV0786	NA	hypothetical protein	−4.7	−3.8
	SAV2553	NA	hypothetical protein	−2.4	−2.2
	SAV0815	*nuc*	staphylococcal nuclease		4.6
	SAV1693	*phoP*	alkaline phosphatase synthesis transcriptional regulatory protein	−2.4	2.6
	SACOL1210	*pyrR*	pyrimidine regulatory protein PyrR	−3	
	SAV1764	*rot*	repressor of toxins Rot		12.6
	SACOL2056	*rsbV*	anti-anti-sigma factor RsbV	−2.9	

Table S1. *Cont.*

Functional Group	Locus ID	Gene	Protein	13136p−m+V5	13136p−m+V20
Regulatory Functions	SACOL0765	*saeS*	sensor histidine kinase	2.1	−2.6
	SACOL0672	*sarA*	staphylococcal accessory regulator A	−2.4	−2.5
	SACOL2287	*sarR*	staphylococcal accessory regulator R	2	
	SACOL0096	*sarS*	staphylococcal accessory regulator S		8.9
	SACOL2258	*sarV*	staphylococcal accessory regulator V	−3.1	
	SAV1835	*traP*	signal transduction protein		2.1
	SACOL1942	*vraR*	DNA-binding response regulator VraR	2.9	
Signal Transduction	SACOL1451	*arlR*	DNA-binding response regulator	−2.5	
	SACOL1450	*arlS*	sensor histidine kinase ArlS	−2.2	
	SAV1692	*phoR*	alkaline phosphatase synthesis sensor protein		2.2
Transcription	SAV0497	NA	translation initiation inhibitor homolog	−3.2	−6.8
	SAV1274	*pnpA*	polyribonucleotide nucleotidyltransferase	−2.1	−2.2
	SACOL1289	*rbfA*	ribosome-binding factor A	2.1	
	SACOL2739	*rnpA*	ribonuclease P	2.1	
	SACOL0588	*rpoB*	DNA-directed RNA polymerase beta subunit		−3.1
	SACOL0589	*rpoC*	DNA-directed RNA polymerase beta' subunit		−2.1
	SACOL2054	*rpoF*	sigma factor B	−2.7	
Transport & Binding Proteins	SAV1696	*aapA*	D-serine/D-alanine/glycine transporter	−2.4	
	SACOL0700	*abcA*	ABC transporter, ATP-binding/permease protein	−2.4	−5.3

Table S1. *Cont.*

Functional Group	Locus ID	Gene	Protein	13136p−m+V5	13136p−m+V20
Transport & Binding Proteins	SACOL1443	*brnQ3*	branched-chain amino acid transport system II carrier protein	2.5	
	SACOL1319	*glpF*	glycerol uptake facilitator protein		−2.1
	SAV0948	*mnhE*	Na+/H+ antiporter subunit	−3	
	SAR0909	*mnhF*	Na+/H+ antiporter subunit	−2.5	
	SAV0946	*mnhG*	Na+/H+ antiporter subunit	−2.3	
	SACOL2272	*modA*	molybdenum ABC transporter, molybdenum-binding protein ModA		3
	SA1972	NA	hypothetical protein	2.7	2.1
	SACOL0261	NA	drug transporter, putative	−2.7	−3
	SACOL0454	NA	sodium:dicarboxylate symporter family protein	3	3.6
	SACOL0630	NA	amino acid permease	−6	−9.5
	SACOL0679	NA	Na+/H+ antiporter, MnhA component, putative	−4.1	−2.5
	SACOL0685	NA	Na+/H+ antiporter, MnhF component, putative	−3.6	−3.7
	SACOL0689	NA	ABC transporter, permease protein	3.7	2.9
	SACOL0690	NA	ABC transporter, ATP-binding protein	2.9	4.4
	SACOL1114	NA	Mn2+/Fe2+ transporter, NRAMP family	−9.5	−9.4
	SACOL1952	NA	ferritins family protein	2.5	3.8
	SACOL2462	NA	ABC transporter, ATP-binding protein	−7.2	−5.4
	SAS0360	NA	putative sodium:dicarboxylate symporter protein	3	4
	SAS0431	NA	sugar-specific PTS transport system, IIBC component	−4.6	

Table S1. *Cont.*

Functional Group	Locus ID	Gene	Protein	13136p−m+V5	13136p−m+V20
Transport & Binding Proteins	SAS1805	NA	hypothetical protein	−4.8	−3.1
	SAV0383	NA	proton/sodium-glutamate symport protein	3.8	4.3
	SAV0626	NA	Na_ antiporter	−4.2	−3
	SAV2185	NA	glycine betaine transporter opuD homolog	−9.2	−14.5
	SAV2301	NA	similar to Na_ antiporter	−3.9	−3.5
	SACOL2292	*nhaC*	Na+/H+ antiporter NhaC	−3.8	−3
	SACOL2721	*nixA*	high-affinity nickel-transport protein		−3
	SAV0986	*oppB*	oligopeptide transport system permease protein	−2.4	
	SACOL0992	*oppC*	oligopeptide ABC transporter, permease protein	−3.1	−2.6
	SACOL0994	*oppF*	oligopeptide ABC transporter, ATP-binding protein	−3.6	−2.4
	SAV2448	*opuCA*	glycine betaine/carnitine/choline ABC transporter		−2.1
	SAR2537	*opuCB*	putative glycine betaine/carnitine/choline transport system permease	−2.6	
	SACOL1384	*opuD1*	osmoprotectant transporter, BCCT family	2.2	
	SAR2276	*opuD2*	glycine betaine transporter 2	−4.9	
	SAV0573	*proP*	proline/betaine transporter homolog	3.7	No Data
	SAV1199	*pyrP*	uracil permease	−2.7	
	SACOL0097	*sirC*	iron compound ABC transporter, permease protein SirC	−7.1	−5.6
	SAV0474	*treP*	phosphoenolpyruvate-dependent and trehalose-specific PTS enzyme II	−4.2	5.3

Table S1. *Cont.*

Functional Group	Locus ID	Gene	Protein	13136p−m+V5	13136p−m+V20
Unclassified	SAV2633	*arcD*	arginine/ornithine antiporter	−2.9	
	SA0742	*clfA*	fibrinogen-binding protein A, clumping factor	−2.2	
	SAV1481	*ebpS*	elastin binding protein	−3.7	
	MW1437	NA	hypothetical protein	−4.6	−7.6
	SAR2275	NA	hypothetical protein	−9.8	−20.4
	SAR2386	NA	putative dehydrogenase	−2.3	−2.3
	SAS0281	NA	hypothetical protein	10	3.6
	SAS1970	NA	serine-protein kinase RsbW	−2.9	−2.8
	SAS2572	NA	hypothetical protein	−3.6	2.7
	SAV0103	NA	similar to Blt-like protein	2.3	6.6
	SAV0277	NA	similar to ABC transporter ATP-binding protein	2.5	2
	SAV0625	NA	MnhD homolog	−2.4	−2.5
	SAV0628	NA	hypothetical protein	−4.3	−3.3
	SAV0681	NA	hypothetical protein	−5.7	−3.9
	SAV0717	NA	similar to urea amidolyase	−4.2	−3.1
	SAV0718	NA	hypothetical protein	−5	−3.7
	SAV1738	NA	hypothetical protein	−3.6	−2.1
	SAV1788	NA	plant metabolite dehydrogenase homolog	2	2.1
	SAV2135	NA	hypothetical protein	−3.4	2.1

Table S1. *Cont.*

Functional Group	Locus ID	Gene	Protein	13136p−m+V5	13136p−m+V20
Unclassified	SAV2366	NA	L-lactate permease lctP homolog	−7.6	3.6
	SAV2473	NA	similar to aminobenzoyl-glutamate transport protein	2.7	3.4
	SAV2565	NA	hypothetical protein	−8.2	−2.3
	SAV2687	NA	hypothetical protein	−2.9	3.1
	SAV1095	*pdhC*	dihydrolipoamide S-acetyltransferase component of pyruvate dehydrogenase complex E2		−3.1
Unknown Function	SACOL1832	*crcB*	crcB protein	3.3	
	SAV1251	*gid*	glucose-inhibited division protein A		−3
	SACOL1191	*mraZ*	hypothetical protein	−2.5	
	SACOL0399	NA	oxidoreductase, putative	−4.6	−4.1
	SACOL0671	NA	hydrolase, alpha/beta hydrolase fold family	−3.9	−3.2
	SACOL1048	NA	acetyltransferase, GNAT family	2.6	2.2
	SACOL1071	NA	chitinase-related protein	2.9	2.1
	SACOL2400	NA	acetyltransferase, GNAT family	2.1	2.5
	SAS2053	NA	haloacid dehalogenase-like hydrolase	2.4	2.1
	SAS2467	NA	putative hydrolase	−7.2	−6.6
	SAV2281	NA	hypothetical protein	2.3	2.1
	SAV2458	NA	hypothetical protein	4.5	2.4
	SAV2474	NA	hypothetical protein	−5.5	−10.8
	SAV2581	NA	conserevd hypothetical protein	−5	−3.4
	SACOL0541	*spoVG*	spoVG protein	−4.6	−5.3
	SACOL1118	*typA*	GTP-binding protein TypA		−5.1
	SACOL1941	*yihY*	predicted membrane protein	−5.3	−4.5

Supplemental Table S2. Expression patterns by gene functional group in VISA 13136p−m+V5 and 13136p−m+V20 as number of genes upregulated and downregulated at least two-fold relative to gene expression in VSSA 13136p⁻m⁺. Values in Unchanged columns indicate numbers of genes with less than a two-fold change in expression between parent and VISA. Data in this table were used to generate Figure 1. A total of 338 genes had expression changes at least two-fold in magnitude in at least one VISA versus parent VSSA; for 335 of these expression data were available for both VISA. No expression data were available for three genes for $13136p^{-}m^{+}V20$: two upregulated in $13136p^{-}m^{+}V5$ (Transport and binding proteins, Cell envelope); one downregulated in $13136p^{-}m^{+}V5$ (Biosynthesis of cofactors, prosthetic groups and carriers).

Gene Functional Group	Genes with Expression Changes	Number of $13136p^{-}m^{+}V5$ Genes			Number of $13136p^{-}m^{+}V20$ Genes		
		85 (25%) Upregulated	188 (56%) Downregulated	65 (19%) Unchanged	80 (24%) Upregulated	177 (52%) Downregulated	78 (23%) Unchanged
Amino acid biosynthesis	12	1	9	2	1	10	1
Biosynthesis of cofactors, prosthetic groups and carriers	18	7	7	4	7	5	5
Cell envelope	48	13	28	7	8	21	18
Cellular processes	11	7	0	4	7	1	3
Central intermediary metabolism	16	1	12	3	3	8	5
DNA metabolism	4	1	2	1	0	3	1
Fatty acid and phospholipid metabolism	3	1	1	1	0	2	1
Mobile and extrachromosomal element functions	1	0	0	1	1	0	0
Protein fate	6	1	1	4	1	5	0
Protein synthesis	27	4	7	16	3	17	7
Purines, pyrimidines, nucleosides and nucleotides	28	4	16	8	1	18	9
Regulatory functions	24	4	15	5	6	11	7
Signal transduction	3	0	3	0	1	0	2
Transcription	7	2	3	2	0	4	3
Transport and binding proteins	39	10	25	4	9	18	11
Hypothetical proteins	50	17	33	0	17	33	0
Unclassified	24	5	18	1	9	12	3
Unknown function	17	7	8	2	6	9	2

Supplementary Table S3. Concordance of gene expression patterns *vs.* parent VSSA 13136p^-m^+ between 13136p^-m^+V5 and 13136p^-m^+V20 for the 335 genes with expression data for both VISA.

83 Genes Overexpressed in 13136p^-m^+V5	Genes	Concordance
Also overexpressed in 13136p^-m^+V20	44	53%
Underexpressed in 13136p^-m^+V20	4	5%
13136p^-m^+V20 expression unchanged	35	42%
**187 Genes Underexpressed in 13136p^-m^+V5**	**Genes**	**Concordance**
Also underexpressed in 13136p^-m^+V20	130	70%
Overexpressed in 13136p^-m^+V20	14	7%
13136p^-m^+V20 expression unchanged	43	23%
**80 Genes Overexpressed in 13136p^-m^+V20**	**Genes**	**Concordance**
Also overexpressed in 13136p^-m^+V5	44	55%
Underexpressed in 13136p^-m^+V5	14	18%
13136p^-m^+V5 expression unchanged	22	28%
**177 Genes Underexpressed in 13136p^-m^+V20**	**Genes**	**Concordance**
Also underexpressed in 13136p^-m^+V5	130	73%
Overexpressed in 13136p^-m^+V5	4	2%
13136p^-m^+V5 expression unchanged	43	24%

Supplementary Table S4. The 45 metabolites with at least one five-fold change among comparisons between 13136p⁻m⁺, 13136p⁻m⁺V5 and 13136p⁻m⁺V20.

Metabolite Class	Metabolite	Metabolite relative concentration per 10 mg dry weight (mean ± SD)			Metabolite Relative Concentration Fold-Change		
		VSSA 13136p⁻m⁺	13136p⁻m⁺V5	13136p⁻m⁺V20	VSSA → V5	VSSA → V20	V5 → V20
Amines & Polyamines	Adenosine-5-monophosphate	ND	ND	5.7 ± 1.2		>100	>100
	Dihydroorotic acid	ND	3.8 ± 0.7	171.8 ± 27.9	>100	>100	45.2
	Glucosamine	ND	6.9 ± 1.5	129.4 ± 30.5	>100	>100	18.8
	Guanine	16.2 ± 1.2	2.7 ± 0.8	1.7 ± 0.3	−5	−10	−1.6
	Guanosine	10.9 ± 2.3	ND	42.7 ± 8.6	<−100	3.9	>100
	Hypoxanthine	ND	ND	0.1 ± 0.0		>10	>10
	Inosine	ND	ND	3.3 ± 0.7		>100	>100
	Nicotinic acid	4.0 ± 0.9	0.5 ± 0.1	9.5 ± 1.5	−8	2.4	19
	Orotic acid	1.7 ± 0.1	1.5 ± 0.1	38.7 ± 1.3	−1.1	23	25.8
	Putrescine	63.3 ± 3.7	63.5 ± 6.5	11.8 ± 1.8		−5.4	−5.4
	Spermidine	42.1 ± 5.7	4.5 ± 0.5	3.7 ± 0.6	−9.4	−11.4	−1.2
	Uracil	23.7 ± 2.1	4.4 ± 0.7	6.5 ± 0.8	−5.4	−3.6	1.5
	Uridine	9.3 ± 1.0	57.6 ± 7.4	13.2 ± 0.9	6.2	1.4	−4.4
Amino Acids	Cystathionine	4.5 ± 0.8	ND	ND	<−100	<−100	
	Glutamic acid	20.5 ± 4.0	284.7 ± 76.8	308.5 ± 36.4	14	15	1.1
	Homoserine	1.0 ± 0.2	ND	0.6 ± 0.1	<5	−1.7	>100
	N-Acetylglutamic acid	ND	ND	25.6 ± 1.5		>100	>100
	O-Acetyl-serine	5.4 ± 1.3	1.0 ± 0.2	ND	−5.4	<−100	<−100
	Ornithine	30.8 ± 4.8	16.6 ± 3.1	1.2 ± 0.2	−2	−26	−13.8
	Proline	10.9 ± 1.5	154.6 ± 30.0	239.8 ± 8.0	14	22	1.6
	Threonine	52.8 ± 10.6	20.0 ± 8.5	7.8 ± 1.2	−2.6	−6.8	−2.6

Table S4. *Cont.*

Metabolite Class	Metabolite	Metabolite relative concentration per 10 mg dry weight (mean ± SD)			Metabolite Relative Concentration Fold-Change		
		VSSA 13136p^{-}m^{+}	13136p^{-}m^{+}V5	13136p^{-}m^{+}V20	VSSA → V5	VSSA → V20	V5 → V20
**Polar Organic Acids**	2-Phosphoglycerate	14.6 ± 2.0	7.5 ± 1.9	3.0 ± 0.6	−2	−5	−2.5
	3-Phosphoglycerate	242.6 ± 45.1	62.7 ± 10.9	40.1 ± 7.5	−3.9	−6	−1.6
	cis-Aconitic acid	ND	2.1 ± 0.2	ND	> 100		<−100
	Citric acid	15.6 ± 2.0	243.7 ± 22.6	35.1 ± 8.4	16	2.3	−6.9
	Fumaric acid	19.8 ± 4.5	1.5 ± 0.4	28.6 ± 4.0	−12.5	1.4	19.1
	Gluconic acid	0.6 ± 0.1	11.8 ± 2.2	5.8 ± 2.1	20	10	−2
	Malic acid	3.4 ± 0.6	ND	12.9 ± 2.3	<−100	3.7	>100
	Pantothenate	ND	ND	2.2 ± 0.0		>100	>100
	Phenylpyruvic acid	ND	ND	3.4 ± 0.4		>100	>100
**Sugars**	Fructose	40.5 ± 7.5	4.0 ± 0.8	1.8 ± 0.2	−10	−23	−2.2
	Galactitol	ND	2.1 ± 0.2	2.0 ± 0.3	>100	>100	−1.1
	Galactose	5.1 ± 1.3	7.9 ± 0.8	1.5 ± 0.3	1.5	−3.3	−5.3
	Glucose-1-P	20.3 ± 5.2	13.8 ± 3.4	2.3 ± 0.6	−1.4	−10	−6
	Glucose-6-P	13.6 ± 0.7	1.6 ± 0.3	0.5 ± 0.1	−8.5	−27.2	−3.2
	Inositol	3.1 ± 1.0	12.8 ± 1.1	0.1 ± 0.0	4.1	−31	−128
	Mannitol	178.1 ± 15.6	29.5 ± 7.2	32.3 ± 3.1	−6	−5.5	1.1
	Mannose	7.2 ± 1.0	12.8 ± 2.4	1.2 ± 0.2	1.8	−6	−10.7
	Mannose-6-P	13.2 ± 2.9	ND	ND	<−100	<−100	
	Ribitol	122.9 ± 22.0	24.2 ± 4.4	76.2 ± 15.4	−5	−1.6	3.1
	Ribose-5-P	4.3 ± 0.5	30.6 ± 1.8	12.5 ± 2.0	7.2	2.9	−2.4
	Sedoheptulose	2.3 ± 0.7	2.2 ± 0.0	ND		<−100	<−100
	Sedoheptulose-7-P	1.0 ± 0.0	ND	ND	<−100	<−100	
	Sucrose	14.5 ± 2.5	1.4 ± 0.7	6.4 ± 0.2	−10	−2.3	4.6
	Trehalose	2.5 ± 0.3	17.6 ± 0.7	3.4 ± 0.8	7	1.4	−5.2

Empty cells: no changes (one-fold changes); ND: Not Detected, with values of 0.01 used for < and > fold-change estimates. Green cells: at least five-fold increase in metabolite concentration; red cells: at least a five-fold decrease in metabolite concentration. Metabolites within each class sorted alphabetically.

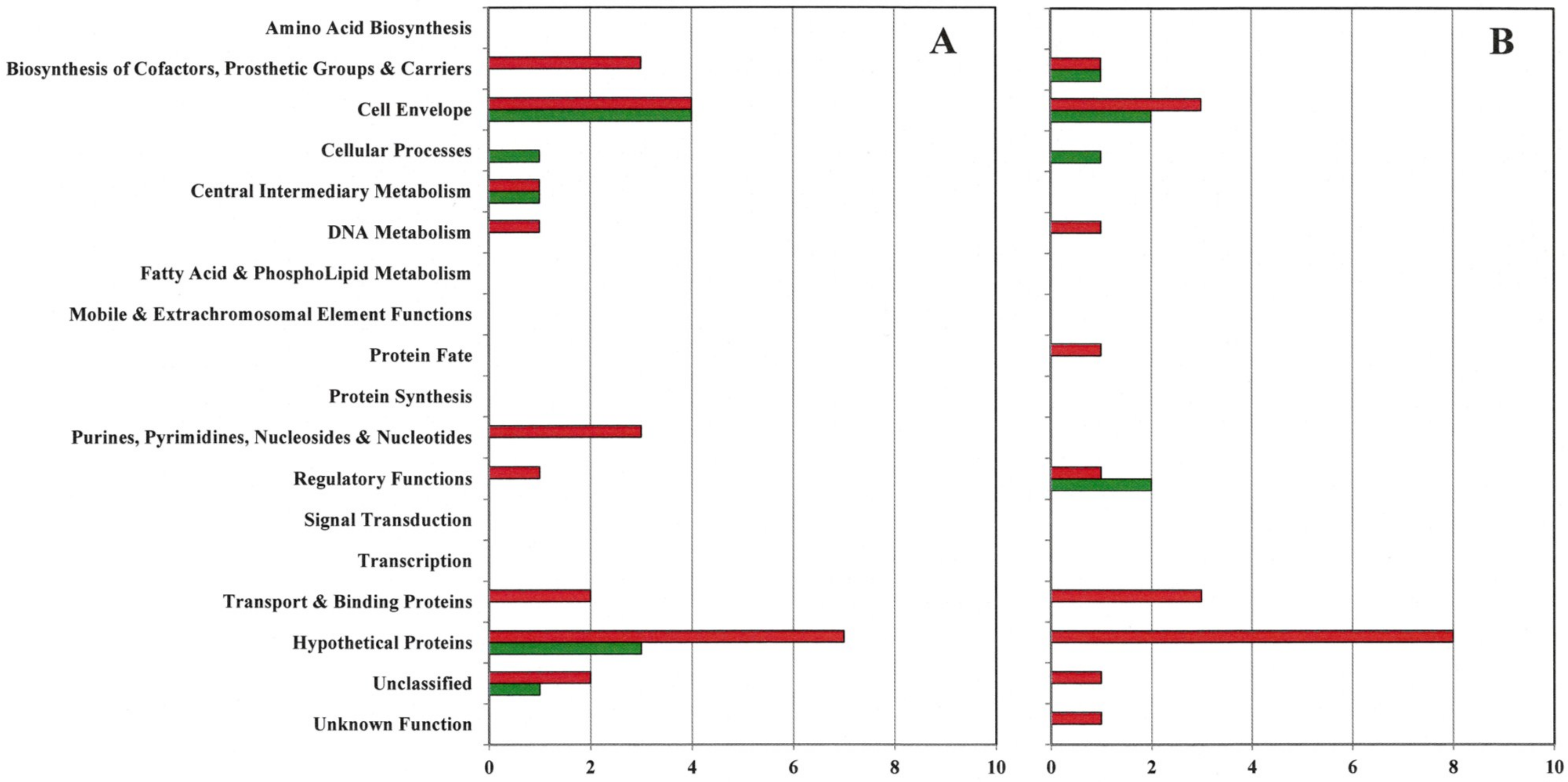

Supplementary Figure S1. Number of genes by functional group upregulated (green bars) and downregulated (red bars) at least eight-fold in VISA 13136p$^-$m$^+$V5 (**A**) and 13136p$^-$m$^+$V20 (**B**) relative to gene expression in VSSA parent 13136p$^-$m$^+$.

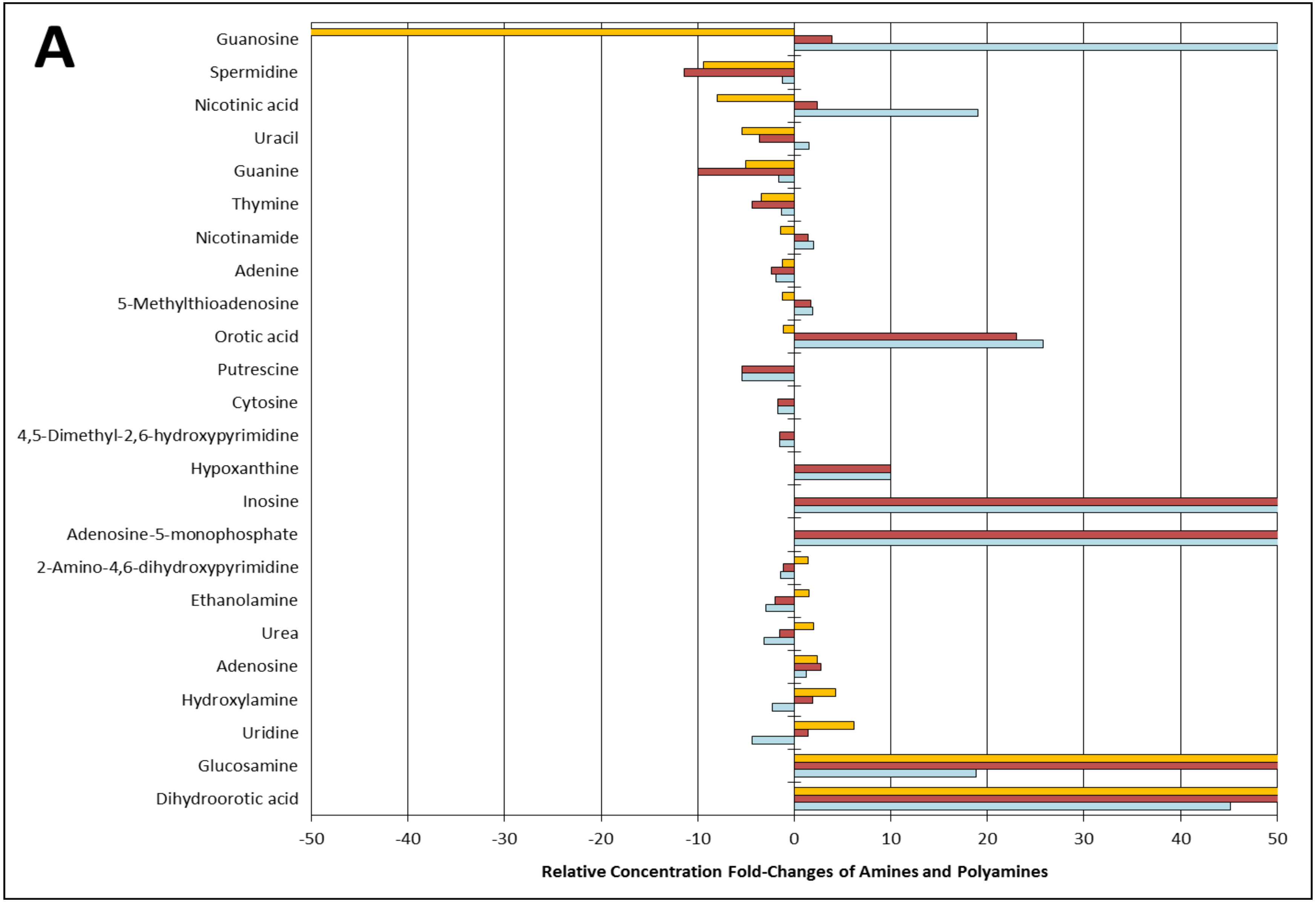

Supplementary Figure S2. *Cont.*

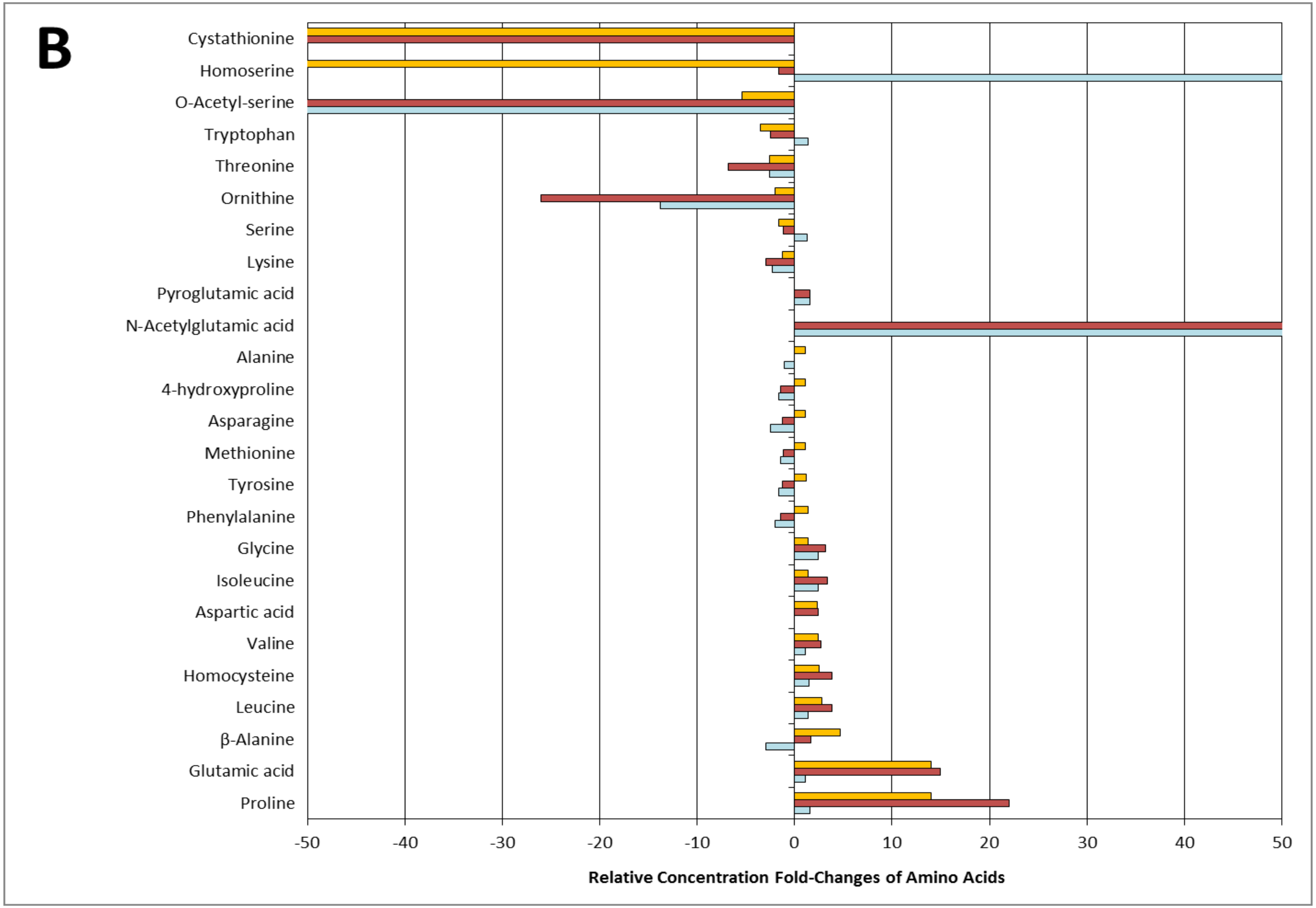

Supplementary Figure S2. *Cont.*

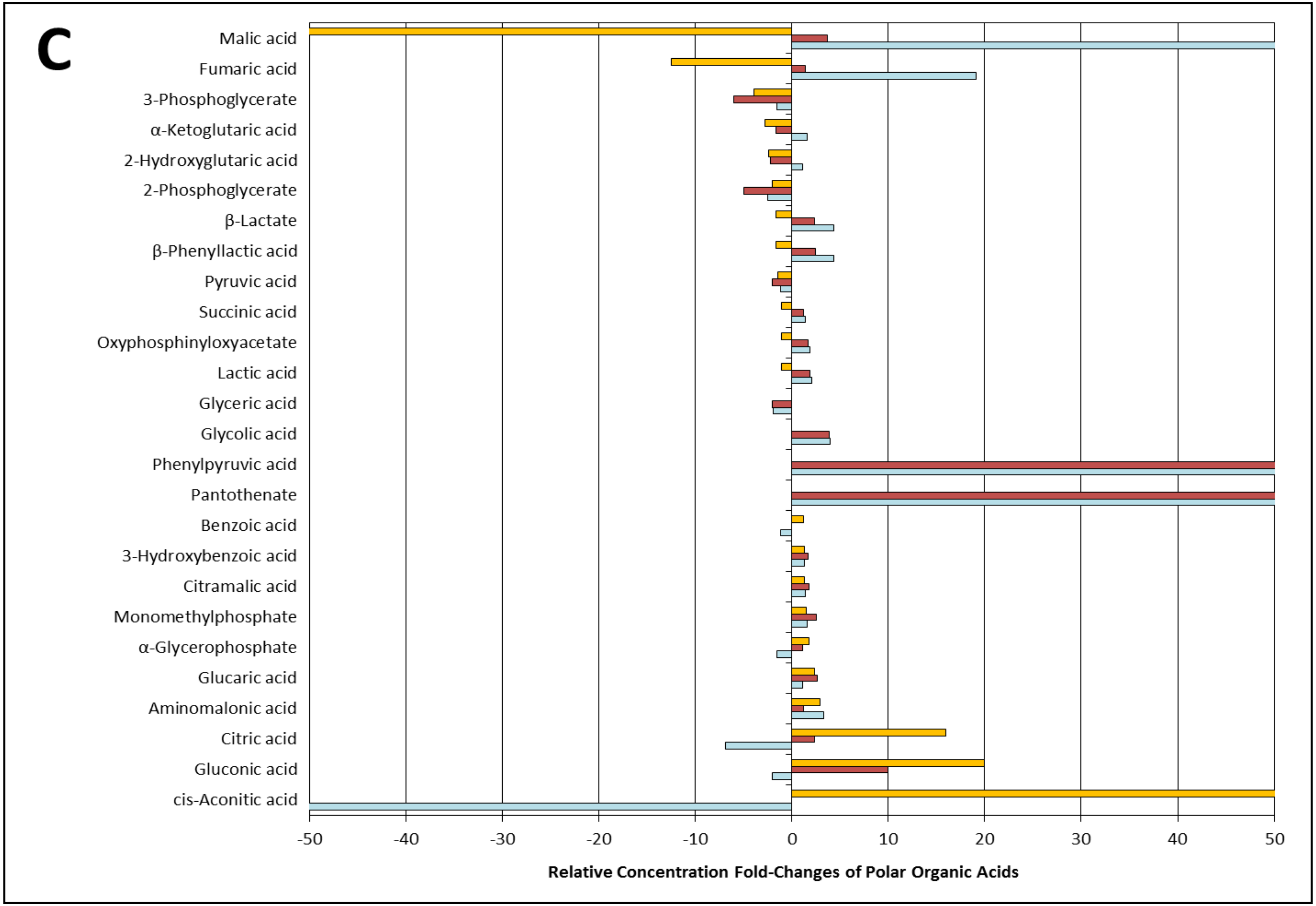

Supplementary Figure S2. *Cont.*

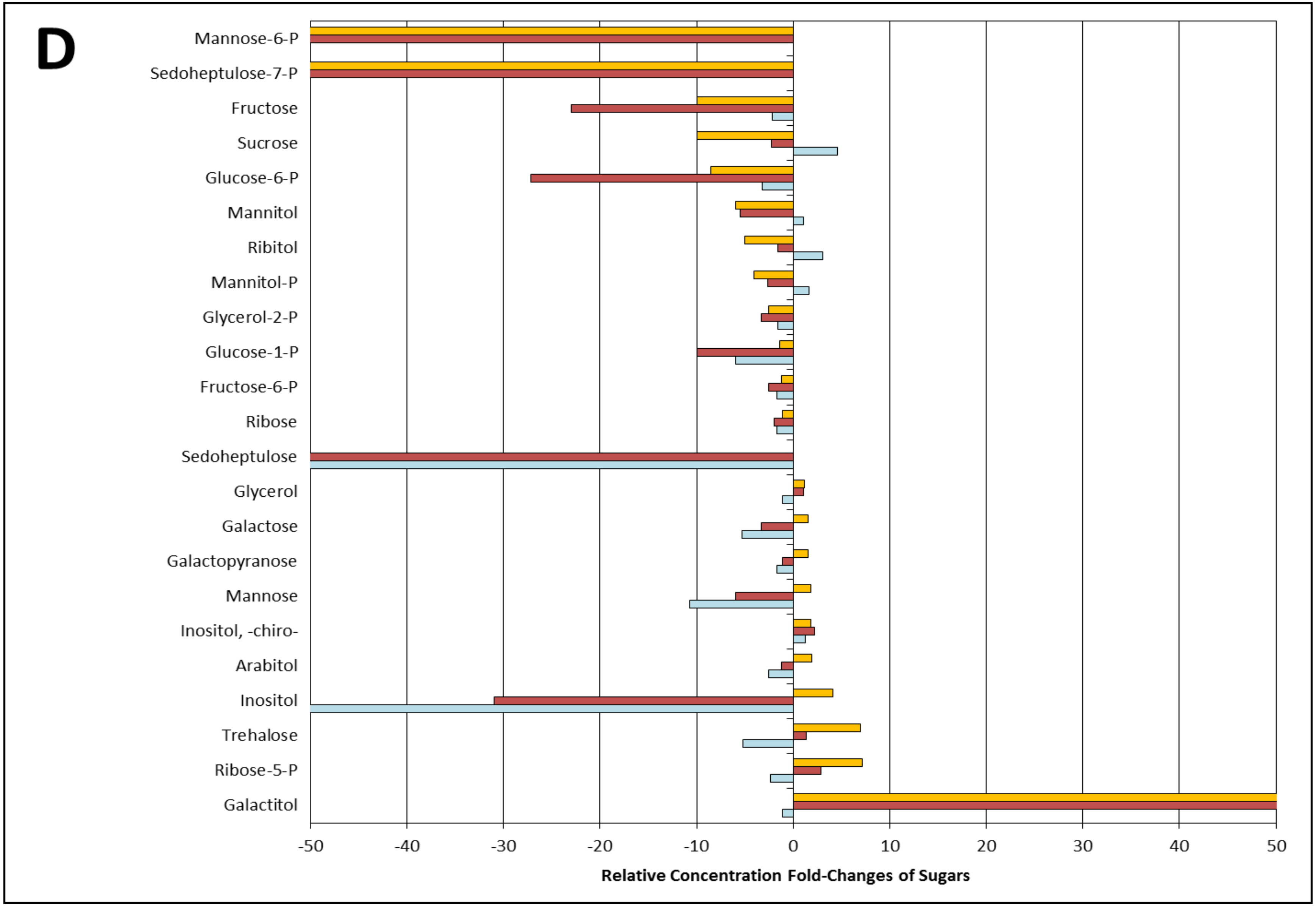

Supplementary Figure S2. *Cont.*

Supplementary Figure S2. Metabolomic profiles by metabolite class (**A**–**D**). Gold bars: metabolite relative concentration changes in VISA 13136p$^-$m$^+$V5 *vs.* VSSA 13136 p$^-$m$^+$. Dark red bars: metabolite relative concentration changes in VISA 13136p$^-$m$^+$V20 *vs.* VSSA 13136 p$^-$m$^+$. Light blue bars: metabolite relative concentration changes in VISA 13136p$^-$m$^+$V20 *vs.* VISA 13136 p$^-$m$^+$V5. Metabolites in each class listed from largest decrease to largest increase in relative concentration fold-change for VISA 13136p$^-$m$^+$V5 *vs.* VSSA 13136 p$^-$m$^+$. All bars extending to x-axis boundaries represent < 100- or > 100-fold changes except inositol in Fig. 2D, for which the relative concentration in VISA 13136p$^-$m$^+$V20 was 128-fold lower than that in VISA 13136p$^-$m$^+$V5.

Supplementary File S1. Metabolomic analysis background information.

The instrument variability was 5%, which is within the standard acceptance limit. Chemometric models were obtained using internal standard-normalized, centered and scaled to Pareto variance data. Obtained metabolite concentrations were analyzed with SIMCA P+ (12.0) software and Metaboanalyst 2.0 (http://www.metaboanalyst.ca) [1]. Missing values were imputed with the ½ of observed minimum positive detection value, assuming their level was below the instrument detection limit. All spurious metabolites derived from column bleed and reagent artifacts were also removed from the data sets.

GC/MS analysis of polar metabolites detected a total of ~1850 EI mass spectral features per sample. Of these, 98 compounds were positively identified as amines and polyamines (24% of total identified compounds), amino acids (26%), carbohydrates (23%) and organic acids (27%).

To analyze the overall levels of identified metabolites within and between strains, box plot analysis of metabolite levels was performed. All samples showed acceptable ranges of metabolite levels and degree of variation within each subgroup (Figure 1).

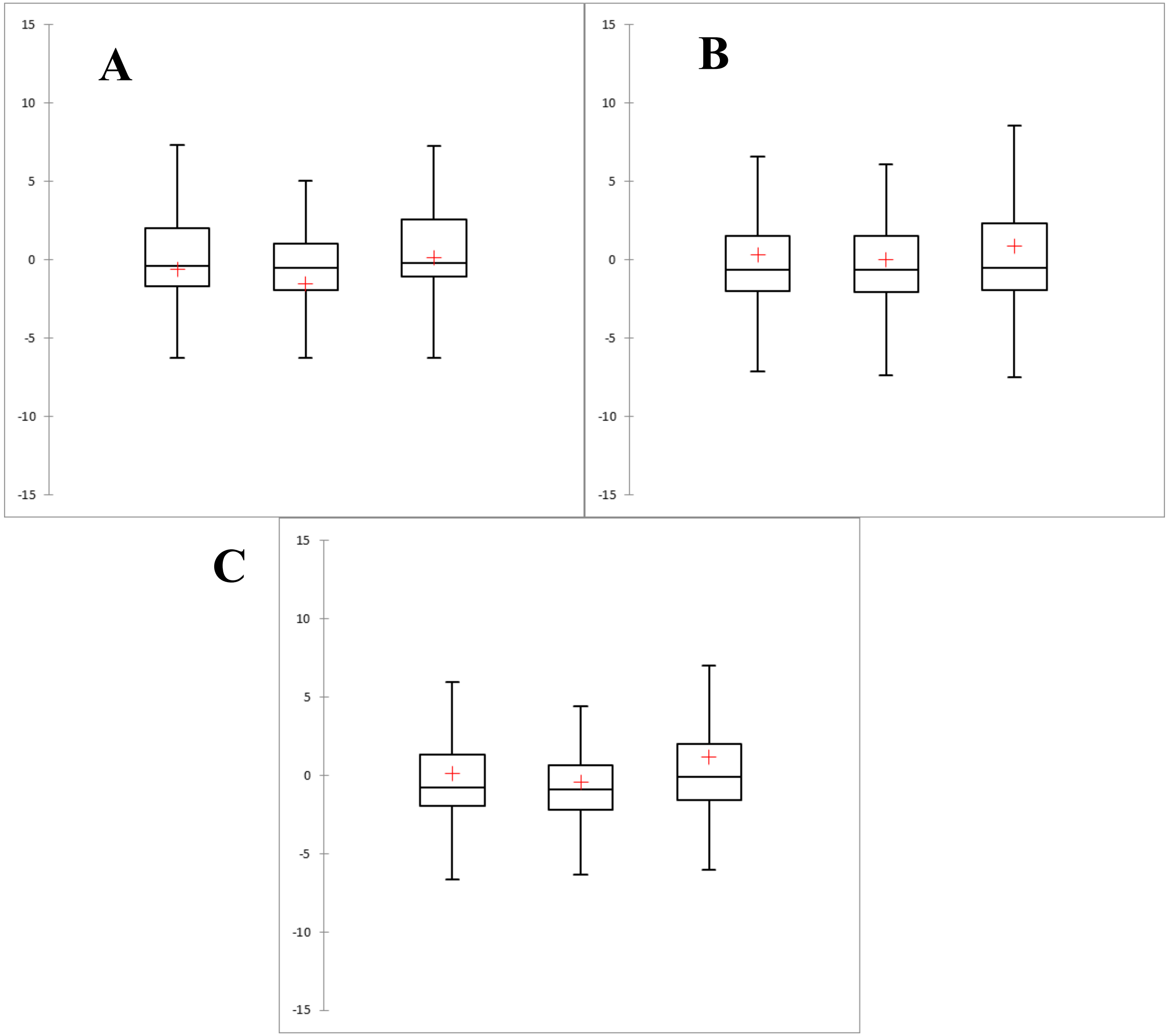

Figure 1. Box plot analysis of metabolite levels within each strain: **A** – *13136p⁻m⁺*, **B** – *13136p⁻m⁺V20*, **C** – *13136p⁻m⁺V5*.

<u>Validation of chemometric models.</u>

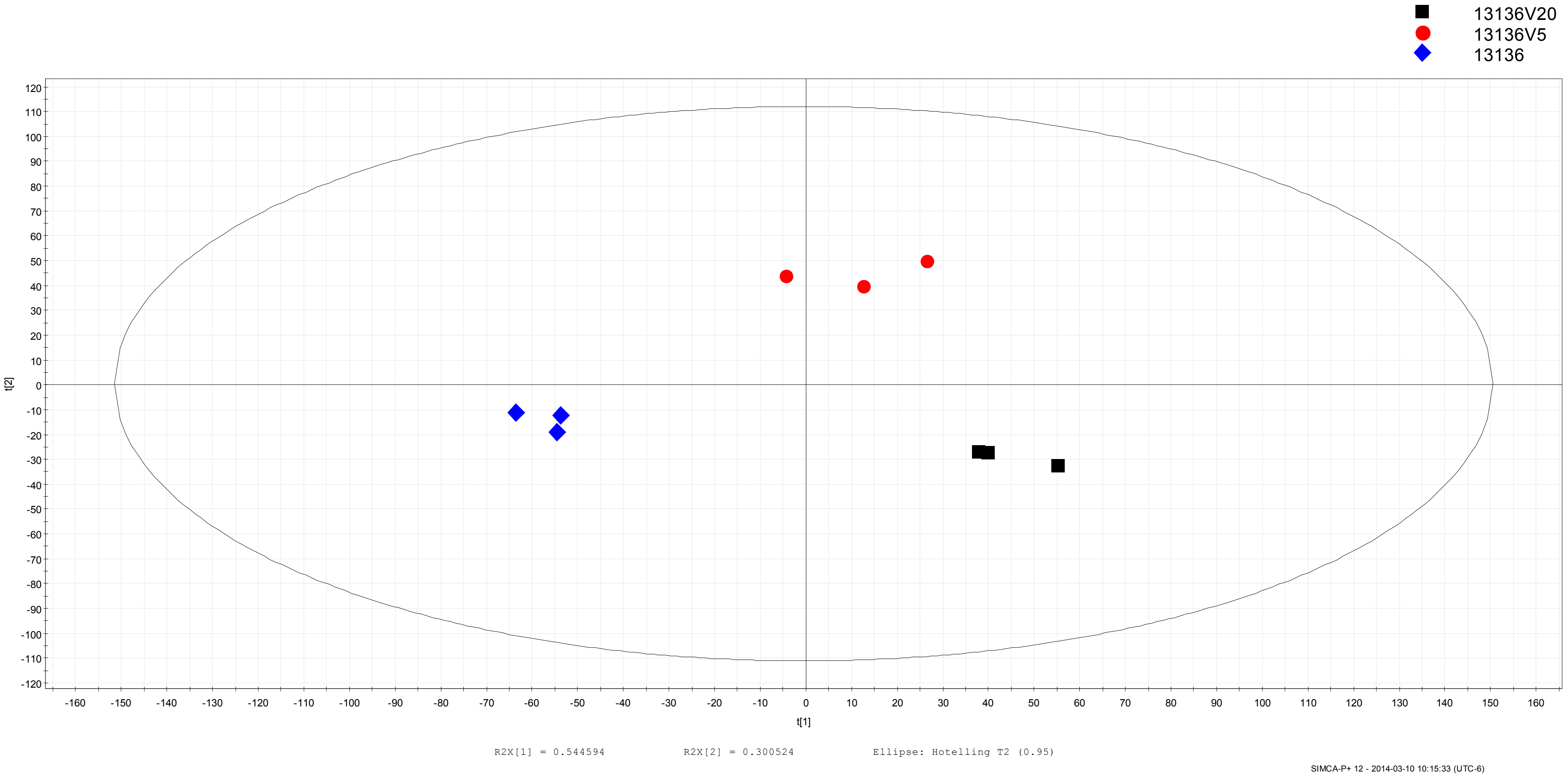

Figure 2. PCA scores plot separating *Staphylococcus aureus* strains. The first component (54% of total variability) distinguished between parent and VISA strains while 13136p$^-$m$^+$V5 and 13136p$^-$m$^+$V20 strains were discriminated along the second component (30% of total variability).

As the number of observations used in chemometric models was much lower than the total number of variables (metabolites), it is possible that the good performance statistics observed in the chemometric models can be due to overfitting of data or chance correlation [2–4]. Therefore, the validity of each model must be evaluated. The PLS-DA model obtained with SIMCA P+ (v. 12.0) software was further analyzed using analysis of variance of sevenfold Cross-Validation predictive residual (CV-ANOVA) and permutation with 300 random reclassifications. Cross-Validation (CV) was used to determine the sufficient number of Principal Components (PCs) represented by the total amount of explained X variance (R2X), Y-variance (R2Y) and cross-validated predictive ability (Q2Y). The PLS-DA model had R2Y = 99.0%, Q2 = 95.9% and variables explained 91.0% (R2X) of total variation. Figure 3 demonstrates a permutation test results—the goodness of fit and predictive ability (R^2/Q^2) of the model. Permutation test summary: A PLS-DA model robustness was assessed by a 300 random permutations ($p < 0.05$) of class membership.

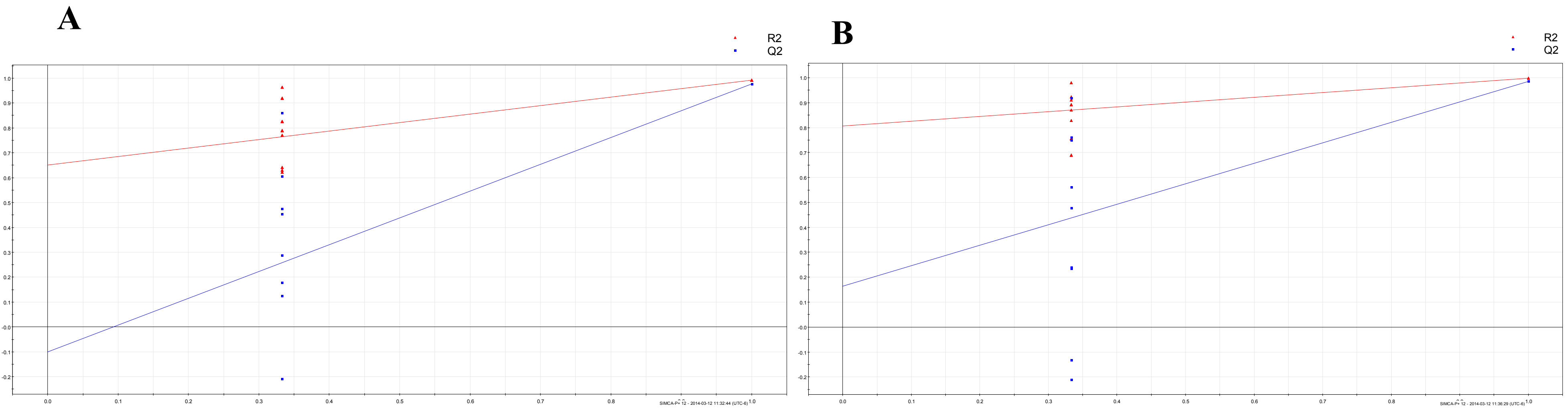

Figure 3. A permutation test of 3 strains (*$13136p^{-}m^{+}V5$, $13136p^{-}m^{+}V20$ and $13136p^{-}m^{+}$*): A - *$13136p^{-}m^{+}V5$, vs. $13136p^{-}m^{+}$*; B - *$13136p^{-}m^{+}V20$ vs. $13136p^{-}m^{+}$*. The vertical axis gives the R2 (red) and Q2 (blue) -values of the original model (far to the right) and the Y-permuted models further to the left. The horizontal axis shows the correlation between the permuted y-vectors and the original y-vector for the selected y.

The criteria for model validity are as follows. First, all the Q^2 values on the permuted data set must be lower than the Q^2 value on the actual data set. If this is not the case, it indicates that the model is capable of overfitting data. The regression line in validation plot (line joining the actual Q^2 point to the centroid of the cluster of permuted Q^2 values) must demonstrate a negative intercept on the *y* axis [5,6].

Evaluation of the discriminating metabolites toward the clustering in PLS-DA model was analyzed using regression coefficient plot with 95% jackknifed confident intervals where metabolites with Variable Importance for Projection (VIP) where scores exceeding 1.5 were selected as important and/or putative biomarkers [7] (Figure 4). VIP is a weighted sum of squares of the PLS loadings that takes into account the amount of explained Y-variance of each component.

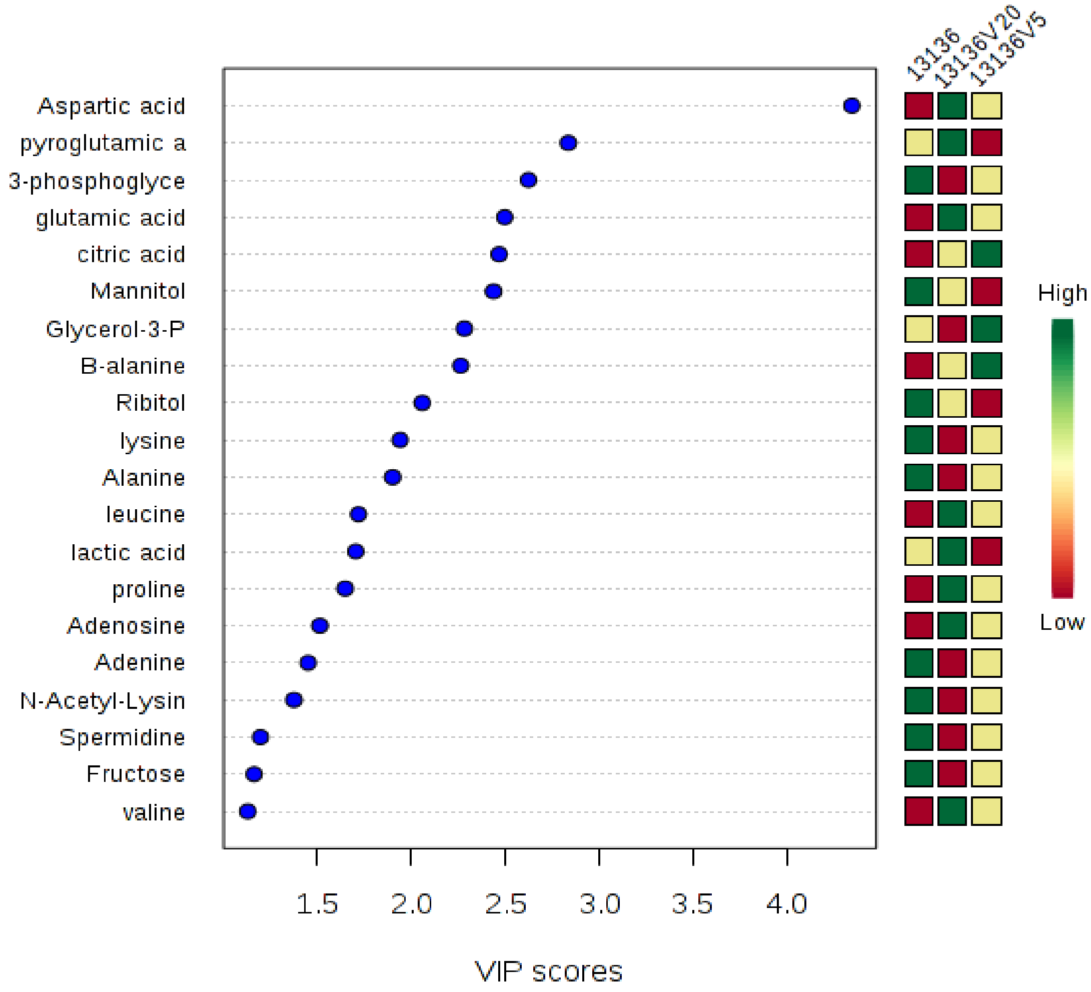

Figure 4. The top 20 compounds ranked by VIP scores in *13136p⁻m⁺V5, 13136p⁻m⁺V20* and *13136p⁻m⁺* strains.

To determine if the group of "important metabolites" (VIP) could classify samples, a Random Forest analysis was performed. The metabolites had a 100% predictive accuracy (Table 1). Thus, the metabolites that are differentially expressed between the groups may represent potential biomarkers for strain discrimination.

Table 1. Results of Random Forest analysis for sample classification by metabolites identified as important by VIP scores.

	**Members**	**Correct**	**13136V20**	**13136V5**	**13136**
13136p⁻m⁺V20	3	100%	**3**	0	0
13136p⁻m⁺V5	3	100%	0	**3**	0
13136p⁻m⁺	3	100%	0	0	**3**
Total	9	100%	3	3	3

References for Supplementary File S1

1. Xia, J.; Wishart, D.S. Web-based inference of biological patterns, functions and pathways from metabolomic data using MetaboAnalyst. *Nat. Protoc*. **2011**, *6*, 743–760.
2. Westerhuis, J.A.; Hoefsloot, H.C.J.; Smit, S.; Vis, D.J.; Smilde, A.K.; van Velzen, E.J.J.; van Duijnhoven, J.P.M.; van Dorsten, F.A. Assessment of PLSDA cross validation. *Metabolomics* **2008**, *4*, 81–89.
3. Smit, S.; van Breemen, M.J.; Hoefsloot, H.C.; Smilde, A.K.; Aerts, J.M.; de Koster, C.G. Assessing the statistical validity of proteomics based biomarkers. *Anal. Chim. Acta* **2007**, *592*, 210–217.
4. Trygg, J.; Holmes, E.; Lundstedt, T. Chemometrics in metabonomics. *J. Proteome Res.* **2007**, *6*, 469–479.
5. Mahadevan, S.; Shah, S.L.; Marrie, T.J.; Slupsky, C.M. Analysis of metabolomic data using support vector machines. *Anal. Chem*. **2008**, *80*, 7562–7570.
6. Chan, E.C.Y.; Pasikanti, K.K.; Nicholson, J.K. Global urinary metabolic profiling procedures using gas chromatography-mass spectrometry. *Nat. Protoc*. **2011**, *6*, 1483–1499.
7. Wiklund, S.; Johansson, E.; Sjöström, L.; Mellerowicz, E.J.; Edlund, U.; Shockcor J.P.; Gottfries J.; Moritz T.; Trygg J. Visualization of GC/TOF-MS-based metabolomics data for identification of biochemically interesting compounds using OPLS class models. *Anal. Chem*. **2008**, *80*, 115–122.

© 2015 by the authors; licensee MDPI, Basel, Switzerland. This article is an open access article distributed under the terms and conditions of the Creative Commons Attribution license (http://creativecommons.org/licenses/by/4.0/).
